# Coral-inspired immunoreprogramming scaffold reverses the “immune-freeze” microenvironment to promote bone regeneration in steroid-induced osteonecrosis of the femoral head

**DOI:** 10.1038/s41413-026-00557-x

**Published:** 2026-06-30

**Authors:** Yue Luo, Qianhao Li, Changjun Chen, Xin Zhao, Zhouyuan Yang, Donghai Li, Ke Jiang, Yan Xiong, Meng Tian, Pengde Kang

**Affiliations:** 1 Department of Orthopedic, West China Hospital, Sichuan University, Chengdu, Sichuan China; 2 Department of Orthopaedics, Affiliated Hospital of North Sichuan Medical College, No. 1 the South of Maoyuan Road, Nanchong, Sichuan China; 3 Department of Orthopedic Surgery, The First Affiliated Hospital of Shandong First Medical University & Shandong Provincial Qianfoshan Hospital, Jinan, China; 4 Department of Orthopedic Surgery, Shandong Provincial Hospital Affiliated to Shandong First Medical University, Jinan, China; 5 Department of Orthopaedics, Daping Hospital, Army Medical University (Third Military Medical Univsersity), Chongqing, China; 6 Department of Neurosurgery and Neurosurgery Research Laboratory, West China Hospital, Sichuan University, Chengdu, Sichuan China

**Keywords:** Bone, Bone quality and biomechanics

## Abstract

The core pathological mechanism of steroid-induced osteonecrosis of the femoral head (SONFH) is an “immune freeze” microenvironment—where corticosteroids continuously drive macrophages toward a pro-inflammatory M1 phenotype, inhibiting the conversion to reparative M2 macrophages, thereby impairing bone regeneration. To address this bottleneck, this study was inspired by the hierarchical pore structure of coral. Using low-temperature deposition 3D printing technology, we constructed an immunoreprogramming biomimetic scaffold that integrates multi-walled carbon nanotubes (MWCNT) and nano-hydroxyapatite (nHA) (MWCNT bionic scaffold). The scaffold leverages the active immune regulatory function of MWCNT to activate the PI3K-AKT signaling pathway, driving macrophages to transition from the M1 to M2 phenotype, effectively breaking the “immunological freeze” state and reshaping the pro-regenerative bone immune microenvironment. Meanwhile, the nHA component provides a biomimetic mineralization matrix and sustained release of calcium and phosphate ions, synergizing with the nanofiber structure of MWCNT to promote the migration and osteogenic differentiation of bone marrow mesenchymal stem cells (BMSCs) and angiogenesis. In vivo experiments confirm that scaffold implantation reverses the local “immune freeze” state, drives macrophage polarization toward the M2 phenotype, reduces inflammatory responses, and enhances the maturation and functional vascular network formation of new bone matrix in bone defect areas, ultimately achieving bone structural reconstruction. The coral-inspired immunoreprogramming strategy proposed in this study provides a new strategy for targeting the regulation of the pathological microenvironment in SONFH.

## Introduction

Steroid-induced osteonecrosis of the femoral head (SONFH) is one of the leading causes of non-traumatic osteonecrosis, and glucocorticoid(GC)-induced osteonecrosis has been reported to occur in approximately 9%–40% of patients receiving corticosteroid therapy, particularly under long-term and/or high-dose exposure.^1^ The core pathological manifestation is GC-induced bone marrow microcirculatory dysfunction, leading to polarization and stagnation of M1-type macrophages and imbalance in the bone immune microenvironment, which in turn triggers ischemic necrosis of osteocytes and progressive joint collapse.^2^ Although core decompression is the gold standard for early intervention, GCs continuously drive macrophages toward the pro-inflammatory M1 phenotype and inhibit their conversion to the reparative M2 phenotype, forming a unique “immune frozen” microenvironment that significantly impedes new bone formation after surgery.^3–5^ Here, we define the ‘immune freeze’ microenvironment as a glucocorticoid-driven pathological immune state characterized by persistent M1 polarization and impaired M1-to-M2 transition, which locks the local milieu in a pro-inflammatory phase and consequently hampers angiogenesis–osteogenesis coupling and effective bone regeneration. We introduce this concept to emphasize that, in SONFH, breaking this immune ‘stagnation’ is a prerequisite for successful post-decompression repair, and thus an immunoreprogramming scaffold that restores M2-skewed resolution represents a rational therapeutic strategy.^3–5^ This microenvironment triggers three pathological effects: impaired angiogenesis, osteoblast-osteoclast coupling dysfunction, and progressive bone structural collapse.^4^ GCs simultaneously inhibit osteogenic differentiation and promote fat infiltration, leading to difficult-to-heal defects in bone tunnels after medullary decompression. Ultimately, approximately 45% of patients require joint replacement, affecting approximately 2.1 million patients worldwide each year and resulting in a significant medical burden.^5,6^

The current clinical gold standard (autologous/allogeneic bone transplantation) faces bottlenecks such as donor shortages, immune rejection, and the inability to regulate the local immune microenvironment.^7^ Emerging biomaterials also have significant drawbacks, primarily manifested as functional singleness (i.e., most materials provide only one major function such as mechanical support or osteoconduction), immunological inertia (i.e., the material behaves in an immunologically passive manner and does not actively engage immune cells toward a pro-regenerative response), and a lack of immunological regulation (i.e., insufficient capability to dynamically modulate the local immune microenvironment during the different phases of bone repair). For example, PLGA/TCP scaffolds containing icariin have osteogenic activity but rely on passive osteoconduction and cannot reverse the imbalance in M1/M2 polarization mediated by GCs^8^; materials such as polyetheretherketone (PEEK) require complex surface modification and neglect immune regulation^9^ and novel black phosphorus scaffolds activate the PI3K-AKT pathway to promote bone differentiation without targeting SONFH immune metabolic deadlock.^10^ Essentially, although existing materials have bone-promoting activity, they cannot actively reverse the “immune freeze” microenvironment induced by GCs, making it difficult to overcome the key bottleneck in SONFH regeneration and repair.

Biologically active ceramics, especially hydroxyapatite (HA), are widely studied materials in bone tissue engineering due to their excellent biocompatibility and bone bonding ability. Among them, nano-hydroxyapatite (nHA) exhibits excellent biocompatibility, osteoconductivity, and osseointegration potential due to its chemical composition, crystal structure, and nanomorphology, which are highly similar to those of natural bone minerals.^11^ Nanotechnology significantly increases the specific surface area and surface energy of nHA, thereby enhancing its biological activity, optimizing its degradation behavior, and improving its bone-inducing capacity. Compared to other nanomaterials (such as metal oxide nanoparticles with ion toxicity risks and polymer nanoparticles lacking bone conductivity), the calcium and phosphorus ions released during the degradation of nHA can directly participate in new bone mineralization. nHA not only provides biomimetic mineral components and nanostructures to promote cell adhesion and differentiation, but the calcium and phosphorus ions released during degradation directly participate in the mineralization of new bone matrix, making it a core bioactive component of bone scaffolds. However, the inherent mechanical performance defects (high brittleness, low toughness) and extremely slow degradation rate of traditional bulk HA are the primary limitations to its application.^12^ Although the nanotechnology of nHA has partially improved its performance, it is still difficult to effectively reverse the “immune freeze” pathological microenvironment of SONFH as a single-component scaffold.

In the design of bone tissue engineering scaffolds, precise regulation of the immune microenvironment is critical for bone regeneration, particularly in complex pathological contexts such as SONFH. The core pathological mechanism of SONFH involves glucocorticoid (GC)-induced bone marrow microcirculatory dysfunction, leading to the polarization and stagnation of pro-inflammatory M1 macrophages and an imbalance in the bone immune microenvironment, which in turn triggers ischemic necrosis of osteocytes and progressive joint collapse.^3–5^ Macrophage phenotype plays a decisive role in inflammation regulation, necrotic tissue clearance, angiogenesis, and stem cell behavior during this process.^3–5^ However, traditional biomaterials (such as single-component nHA scaffolds) and the clinical gold standard intervention, core decompression surgery, are unable to effectively reverse the unique pathological immune state driven by GCs; Although core decompression can alleviate intraosseous pressure, it cannot resolve the persistent M1 polarization induced by GCs and its significant inhibition of the conversion to the reparative M2 phenotype, thereby forming and maintaining an “immune frozen” microenvironment, leading to insufficient new bone formation postoperatively.^4,13^ Therefore, developing bone repair materials with active “immune reprogramming” functions to break this frozen state and transform the pro-inflammatory microenvironment into a pro-regenerative microenvironment has become a key strategy.^7^ Multi-walled carbon nanotubes (MWCNTs) exhibit significant immunomodulatory potential due to their unique physicochemical properties. They can effectively intervene in GCs-mediated macrophage abnormal polarization, reverse M1 stagnation, and promote its conversion to the M2 phenotype that promotes repair and angiogenesis, thereby directly targeting the core mechanism of “immune freezing” in SONFH.^14–16^ Additionally, MWCNTs possess high electrical conductivity and have been widely incorporated into electroactive scaffolds; such conductive components may facilitate charge transfer and provide electroactive cues, which have been reported to support osteogenic differentiation and angiogenic responses in bone regeneration studies.^17,18^ Their large specific surface area can serve as a delivery vehicle for anti-inflammatory factors (such as IL-4) or pro-angiogenic drugs (such as VEGF). The nanoscale tubular structure can mimic the nanoscale morphology of the extracellular matrix (ECM), synergistically addressing multiple pathological effects associated with “immune freezing”.^19–22^ Given this, integrating MWCNTs with immune reprogramming capabilities into nHA-based biomimetic scaffolds provides a direct pathway for targeting and reversing the core pathological mechanisms of SONFH, particularly GC-induced M1 stasis and “immune freeze.” This integration is expected to synergize with nHA’s excellent bone conduction/bone induction activity with MWCNTs’ immune regulation, high specific surface area, and nanotopological structure properties, creating an “immune reprogramming-type biomimetic scaffold” that can effectively overcome “immune freezing” and promote angiogenesis and bone regeneration, thereby offering a new strategy to improve the therapeutic efficacy of SONFH core decompression surgery.

**Fig. 1:**
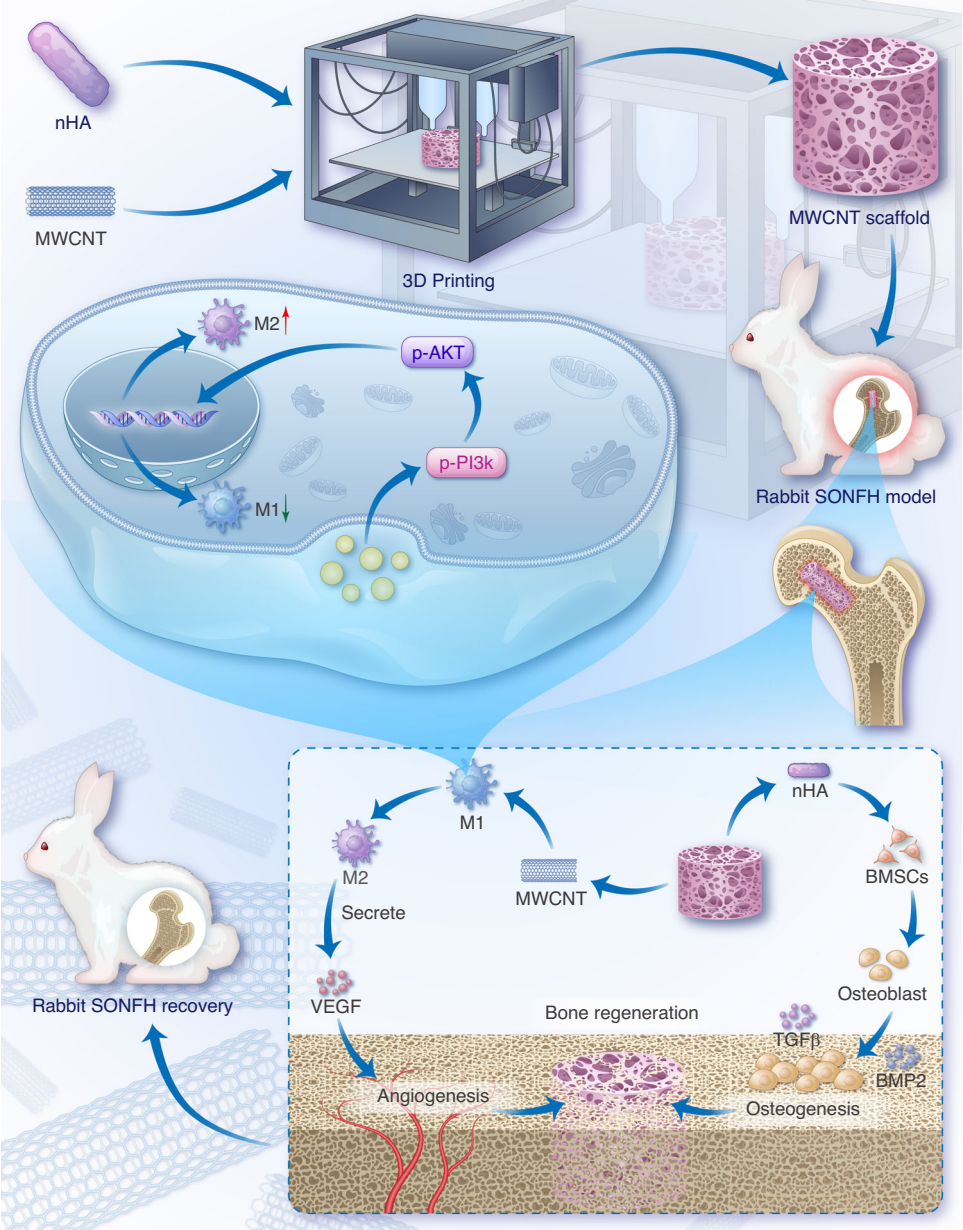
Schematic diagram of a type of biomimetic scaffold (MWCNT bionic scaffold) inspired by the hierarchical pore structure of coral for the treatment of steroid- induced osteonecrosis of the femoral head

Therefore, in response to the dual challenges posed by the “immune freeze” microenvironment hindering bone regeneration in SONFH and the need for ideal bone implants to mimic the natural bone microstructure and mechanical properties, there is an urgent need to develop intelligent bone repair scaffolds that can simultaneously mimic the natural bone microstructure and mechanical properties of natural bone and actively remodel the pathological microenvironment. Our inspiration comes from an ancient marine organism—coral—whose skeletal structure features nested layers of large and small pores, forming natural channels for efficient nutrient and cell transport.^23–25^ Inspired by this, we constructed a layered porous immune reprogramming bionic scaffold (MWCNT bionic scaffold, Fig. 1) integrated with MWCNTs using low-temperature deposition 3D printing technology, aiming to target the repair of SONFH. The core design strategy of this scaffold lies in leveraging the immunomodulating properties of MWCNTs to specifically intervene in the abnormal polarization state of macrophages induced by GCs, reversing the pro-inflammatory M1 phenotype polarization stagnation and promoting its transformation into the reparative M2 phenotype, thereby directly breaking the critical “immune frozen” pathological microenvironment of SONFH. This immune reprogramming effect not only significantly improves the local inflammatory state (reducing pro-inflammatory factors and enhancing anti-inflammatory factor secretion) but also promotes the osteogenic differentiation and migration of BMSCs. Additionally, the nHA in the scaffold provides a biomimetic mineralization microenvironment and sustained release of calcium/phosphorus ions, synergizing with the MWCNTs’ high specific surface area and nano-topological structure characteristics to jointly promote angiogenesis and osteogenesis. In summary, the immune reprogramming-type MWCNT biomimetic scaffold constructed in this study provides a potential therapeutic strategy for reversing the “immune freeze” microenvironment in early SONFH and promoting bone repair.

## Results

### Fabrication and characterisation of MWCNT bionic scaffolds

**Fig. 2:**
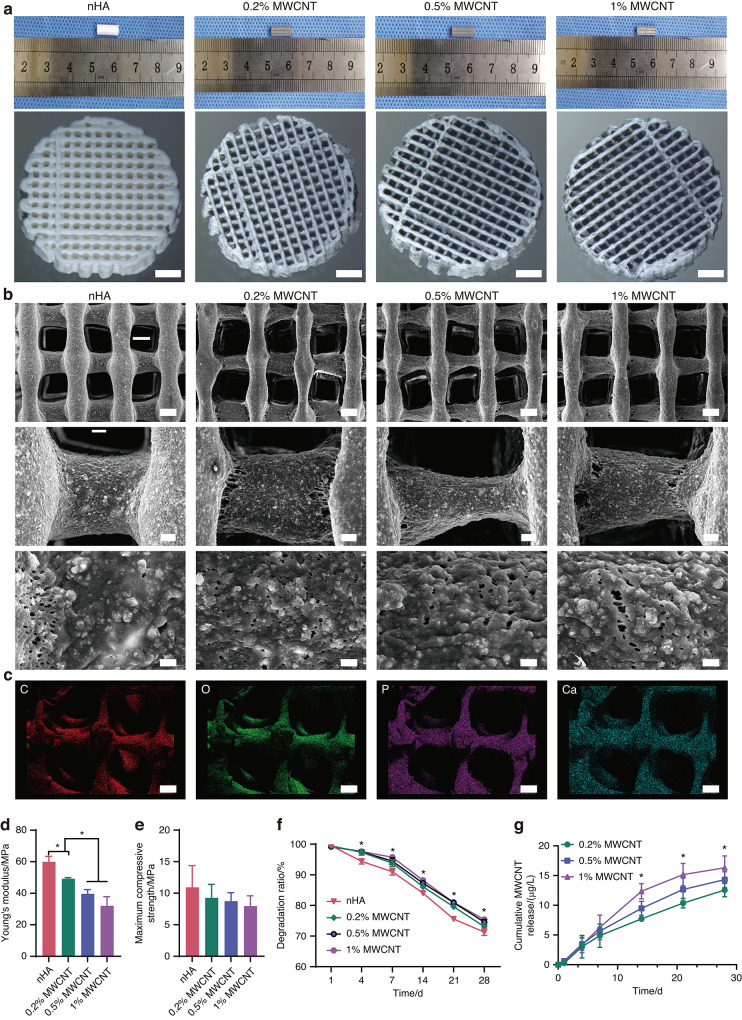
Morphology and characterization of MWCNT bionic scaffolds. **a** Digital images of MWCNT bionic scaffolds of different compositions. All scaffolds share the same orthogonal lattice macro-architecture; only the MWCNT content differs among groups. The scale bar is 1 000 μm. **b** Morphology of MWCNT porous scaffolds observed by SEM. The scale bar is 200 μm, 50 μm and 10 μm in turn. **c** Cross-sections of scaffolds of different compositions were observed using energy dispersive spectroscopy (SEM-EDS). Carbon (C, red); oxygen (O, green); phosphorus (P, purple); calcium (Ca, blue). The scale bar is 100 μm. **d** Young’s modulus of MWCNT bionic scaffolds with different compositions. **e** Compression modulus of MWCNT scaffolds with different compositions. **f** Change in weight of each group of porous scaffolds in PBS solution. **g** Cumulative concentration of MWCNT released from each group of scaffolds in PBS solution. *P < 0.05; **P < 0.01; ***P < 0.001

In this study, inspired by the multi-level pore structure of coral, multi-walled carbon nanotubes (MWCNTs) integrated with nano-hydroxyapatite (nHA) were prepared using low-temperature deposition 3D printing technology to create an immunoreprogramming-type biomimetic scaffold (MWCNT scaffold). Scanning electron microscopy (SEM) analysis (Fig. 2a, b) revealed that all scaffolds exhibited a regular layered porous structure composed of orthogonally arranged filaments.

Notably, all groups shared identical orthogonal lattice geometry, and only the MWCNT content was varied. This hierarchical porous sponge-like structure consists of large and micro pores extensively interconnected across the scaffold, offering advantages over uniformly porous structures,^26^ which is conducive to cell infiltration, nutrient transport, cell adhesion, and bioactive molecule adsorption.^27^ Under high-power SEM, the filament diameter exhibits a honeycomb-like loose porous structure with a slightly rough surface and open microchannels, and the fiber spacing is similar to that of bone trabeculae. Under high-magnification SEM, the filament diameter exhibits a honeycomb-like porous structure with a slightly rough surface and open microchannels, whose fiber spacing is similar to that of trabecular bone. Energy-dispersive X-ray spectroscopy (EDS) elemental distribution maps (Table S1, Figs. 2c and S1a) confirm the presence of O, C, Ca, and P elements in the scaffold, which are uniformly distributed across the filament surface, indicating successful composite formation between nHA and MWCNT. X-ray diffraction (XRD) patterns (Fig. S1b) show that the MWCNT scaffold exhibits characteristic MWCNT peaks at 2θ = 26° (002) and 43° (100),^28^ while retaining the characteristic peak of nHA at 2θ = 31.87° (JCPDS 9-0432),^29^ further confirming the deposition of MWCNT on nHA. As the MWCNT doping concentration increased (0.2% → 1%), the (002) and (100) peak positions shifted toward lower angles (26.46° → 26.03°; 43.63° → 43.21°). Mechanical testing indicated that the Young’s modulus of the 1% MWCNT scaffold (32.01 ± 5.83 MPa) was significantly lower than that of the 0.2% MWCNT (49.19 ± 0.71 MPa) and nHA scaffolds (59.95 ± 3.46 MPa), but there was no significant difference compared to the 0.5% MWCNT scaffold (40.13 ± 3.59 MPa) (Fig. 2d). However, the compressive strength of the scaffolds in each group (nHA: 10.93 MPa; 0.2% MWCNT: 9.25 MPa; 0.5% MWCNT: 8.73 MPa; 1% MWCNT: 7.95 MPa) was not significantly different (Fig. 2e). The compressive strength and modulus of all groups of scaffolds were within the mechanical range of natural cancellous bone (2–12 MPa),^30^ indicating that it has suitable mechanical support properties for bone tissue engineering.

The introduction of MWCNT did not significantly improve the water absorption rate (Fig. S1c) and specific surface area (SSA, Fig. S1e) of the scaffold. Pore size is a key factor affecting cell adhesion, migration, and metabolism.^31^ There were no significant differences in the porosity of the scaffolds among the groups (nHA: 81.24%; 0.2% MWCNT: 79.93%; 0.5% MWCNT: 80.25%; 1% MWCNT: 75.01%) (Fig. S1d). This 3D-printed scaffold features controllable macroporous (approximately 400 μm) and microporous structures. The pore diameter distribution was consistent (nHA: 9.6 ± 1.4 μm; 0.2% MWCNT: 9.2 ± 1.1 μm; 0.5% MWCNT: 9.1 ± 1.3 μm; 1% MWCNT: 9.1 ± 1.4 μm) (Fig. S1f). Pore size distribution analysis (Fig. S2) shows that the main pore sizes are concentrated between 2 and 16 μm, with an average pore size of approximately 9 μm, meeting the requirements of bone tissue engineering for a hierarchical pore structure (large pores >100 μm promote bone ingrowth/nutrient transport, micro pores ~10 μm facilitate capillary formation/cell adhesion, and nano pores facilitate protein adsorption/cell response).^32,33^ This structure effectively mimics the biomimetic characteristics of trabecular bone. In vitro release experiments showed (Fig. 2g) that all three MWCNT scaffolds continuously released MWCNT into the cell culture medium over 28 days, with the 1% MWCNT group exhibiting a significantly higher release rate than the other concentration groups after day 14. In vitro degradation experiments showed (Fig. 2f) that the mass of all scaffolds decreased over time, with the nHA scaffold exhibiting a significantly higher degradation rate than the MWCNT scaffolds, whose mass loss became evident only after 7 days.

### Biocompatibility of MWCNT bionic scaffolds of different compositions

**Fig. 3:**
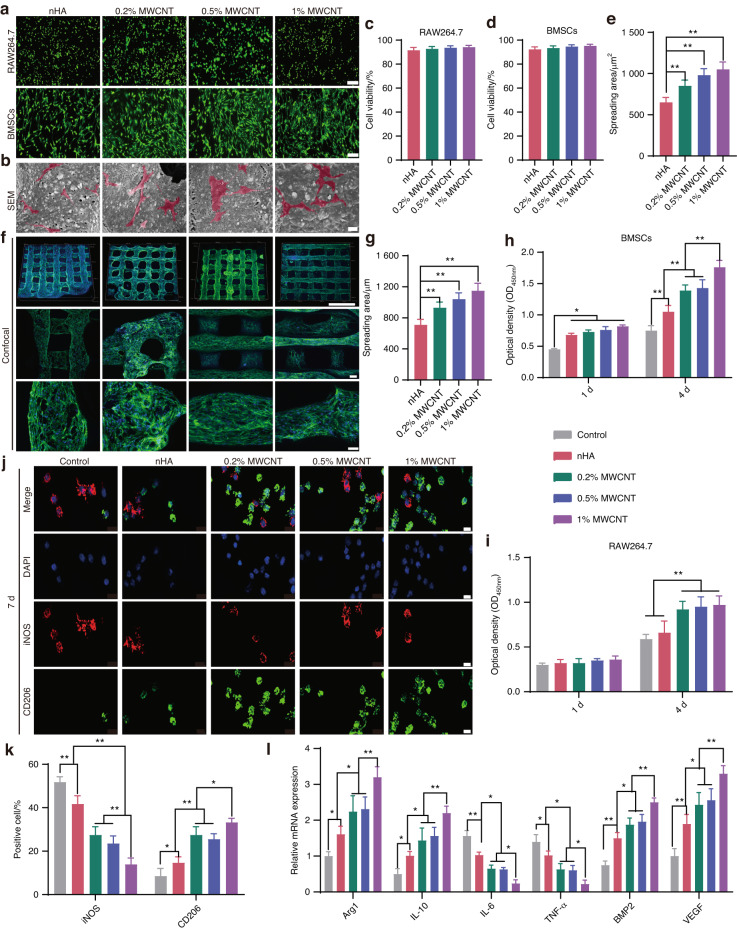
Biocompatibility and macrophage polarization-regulatory properties of the biomimetic scaffolds. **a** Live-dead staining results of rBMSCs and RAW264.7 on the surface of different bionic scaffolds. The scale bar is 200 μm. **b** The changes of cell morphology of rBMSCs on different bionic scaffolds were observed using scanning electron microscopy (SEM). The scale bar is 20 μm. **c**, **d** Quantification of cell viability (%) based on Live/Dead staining images. **e** Quantification of rBMSC spreading area (μm²) based on SEM images. **f** Morphological changes of cells of rBMSCs (actin filaments were stained green while nuclei were stained blue) were observed using confocal laser scanning microscopy (CLSM) when rBMSCs were cultured on different scaffolds for 4 days. The scale bars from top to bottom are 1 000 μm, 100 μm, and 50 μm. **g** Quantification of rBMSC spreading area (μm²) based on confocal images. **h** Cell viability (CCK-8) of rBMSCs on different biomimetic scaffolds on days 1 and 4. **i** Cell survival of RAW264.7 on different scaffolds on day 1 and 4 (CCK-8). **j**, **k** RAW264.7 pretreated with GCs+LPS combined with IFN-γ for 24 h was inoculated onto different scaffolds and cultured, and then subjected to immunofluorescence staining (**j**, CD206 in green; iNOS in red; DAPI in blue) and semi-quantitative analysis of the results (**k**). The scale bar is 10 μm. **l** RAW264.7 pretreated with GCs+LPS combined with IFN-γ for 24 h was inoculated onto different scaffolds and cultured, and then RT-PCR was used to determine the mRNA expression of cytokines related to the M1 and M2 phenotypes of RAW264. 7 cells on different scaffolds. *P < 0.05; **P < 0.01; ***P < 0.001

The biocompatibility of the scaffolds was evaluated by seeding RAW 264.7 cells or rat bone marrow mesenchymal stem cells (rBMSCs) on the scaffold surface. Live-dead staining showed (Fig. 3a) that there was no significant difference in the number of dead cells between the MWCNT scaffold group and the nHA control group, indicating that no obvious cytotoxicity was introduced. SEM observation revealed that rBMSCs in the nHA group exhibited an elongated morphology with limited spreading (limited pseudopod extension), while cells in the MWCNT group exhibited a typical polygonal spreading morphology (Fig. 3b). Cytoskeletal staining (FITC-phalloidin/DAPI) further confirmed that rBMSCs in the MWCNT group had a larger spreading area, richer pseudopod structure, and significantly enhanced F-actin expression (Fig. 3f). To further strengthen the data presentation, we performed quantitative analyses for cell viability and cell spreading on the scaffolds. For Live/Dead staining, the percentage of live cells (green) and dead cells (red) was quantified from at least three randomly selected fields per sample using ImageJ. As shown in Fig. 3c, d, all groups maintained high cell viability (>90%), indicating negligible cytotoxicity after incorporating MWCNTs. In addition, cell spreading was quantified based on both SEM images and confocal F-actin staining. The projected spreading area of rBMSCs was measured using ImageJ, and the results are shown in Fig. 3e, g. The MWCNT-containing scaffolds exhibited significantly increased cell spreading compared with the nHA scaffold, suggesting improved cell adhesion and cytoskeletal organization induced by the MWCNT nanotopography. The CCK-8 proliferation assay quantitatively demonstrated that the MWCNT scaffold significantly enhanced the cell viability of RAW 264.7 and rBMSCs (*P < 0.05 vs. nHA group), with the 1% MWCNT group showing the most significant proliferative effect (Fig. 3h, i). Highly spread polygonal morphology and abundant F-actin expression are key morphological markers of osteogenic differentiation. MWCNT scaffolds promote osteogenic differentiation of rBMSCs by regulating cell spreading behavior and cytoskeletal reorganization.

### Immunomodulation of macrophage phenotypic transformation under glucocorticoid action by MWCNT bionic scaffolds of different compositions

Macrophages are key regulators of early inflammatory control and bone homeostasis in bone regeneration,^10^ regulating the immune microenvironment through phenotypic plasticity. In SONFH, M1 macrophages are continuously activated and unable to polarize toward the M2 type, leading to chronic inflammation and impaired bone repair.^2,34^ This work stimulated the formation of an M1 macrophage model that mimicked the inflammatory milieu of SONFH using interferon-γ + lipopolysaccharide + dexamethasone (Fig. S3, S4). Immunofluorescence analysis showed (Fig. 3j, k) that the proportion of M1 phenotype (iNOS^+^) was significantly increased in the control group, the expression of M2 markers (CD206^+^) was significantly increased in the MWCNT scaffold group compared with the nHA group, and the expression of M1 markers (iNOS) in the MWCNT group was significantly lower than that in the nHA group (*P < 0.05), with the 1% MWCNT group exhibiting the optimal phenotypic regulatory effect (CD206^+^ peak/iNOS^+^ trough). The above results indicate that the MWCNT scaffold effectively alleviates local inflammation by promoting macrophage polarization toward the M2 phenotype, thereby creating a favorable immune microenvironment for bone regeneration.

RT-PCR analysis confirmed that macrophage phenotype regulates the bone repair microenvironment through cytokine secretion.^35^ RT-PCR analysis revealed (Fig. 3l): The MWCNT scaffold group significantly downregulated the expression of M1 phenotype genes (IL-6, TNF-α) (*P < 0.05 vs. nHA group), with the inhibition of TNF-α being particularly critical—it directly impairs bone repair by blocking ALP synthesis and extracellular matrix mineralization. Concurrently, this group upregulated the expression of M2 phenotype genes (Arg-1, IL-10, BMP2, VEGF), with IL-10 inhibiting excessive inflammatory responses and the TGF-β/BMP2 pathway directly driving osteogenesis.^35^ The 1% MWCNT group exhibited the optimal gene regulatory effect. The gene expression profile is consistent with macrophage phenotype conversion data, confirming that MWCNT scaffolds promote M2 phenotype conversion by reversing glucocorticoid-induced M1 polarization, thereby creating an immune microenvironment conducive to bone regeneration.

### MWCNT bionic scaffolds mediate macrophage activation for osteogenic differentiation

**Fig. 4:**
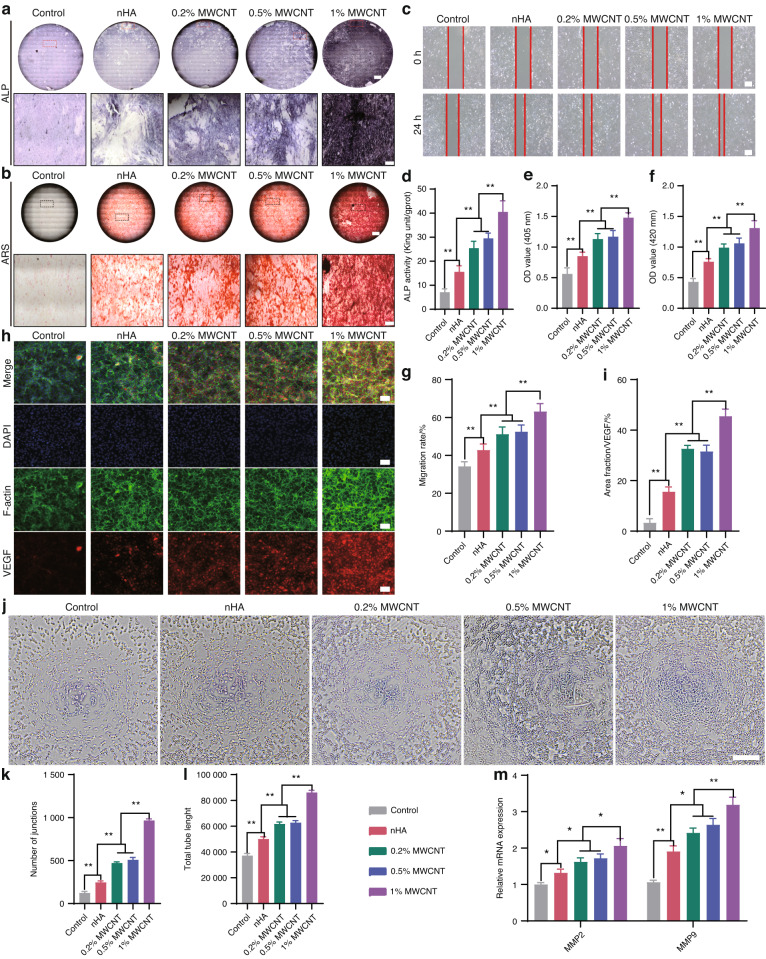
Effects of scaffold-mediated macrophage immunomodulation on osteogenesis and angiogenesis in vitro. **a** ALP staining of rBMSCs grown in the Transwell co-culture system that has been pretreated (various scaffolds co-cultured with RAW264.7 (GCs+LPS combined with IFN-γ pretreatment)). The scale bar is 1 000 μm and 200 μm in turn. **b** rBMSCs grown in the Transwell co-culture system after pretreatment stained with ARS. The scale bar is 1 000 μm and 200 μm in turn. **c** Outcomes of rBMSC scratch tests conducted in the Transwell co-culture system after pretreatment. There is a 100 μm scale bar. **d** rBMSCs grown in the pretreatment Transwell co-culture system were tested for ALP activity. **e** ALP staining quantitative results. **f** ARS staining quantitative results. **g** rBMSC scratch assay cell migration rate analysis. **h** VEGF immunofluorescence staining images in HUVECs (VEGF in red, F-actin in green, and DAPI in blue). There is a 50 μm scale bar. **i** Semi-quantitative analysis of VEGF immunofluorescence. **j**–**l** Tube-forming experiments’ results (**j**) and quantitative evaluation of the total length of tubular patterns and the number of vascular crossings of HUVECs grown in the pretreatment Transwell co-culture system (**k**, **l**). Scale bar: 200 μm. **m** RT-PCR in HUVECs revealed the presence of angiogenesis-related genes (MMP2 and MMP9). *P < 0.05; **P < 0.01; ***P < 0.001

To elucidate the osteogenic-immunomodulatory association mechanism, this study evaluated the immunomodulatory osteogenic effects of MWCNT scaffolds using a macrophage-rBMSC co-culture system. After 7 days of osteogenic induction, ALP staining showed that ALP activity in all MWCNT groups and the nHA group was significantly higher than that in the control group, manifested as increased intracellular blue-purple granular precipitation (Fig. 4a). Quantitative analysis further confirmed that the ALP activity in the 1% MWCNT group was the highest, being 1.4-fold, 1.6-fold, 2.6-fold, and 5.7-fold higher than that in the 0.5% MWCNT, 0.2% MWCNT, nHA, and control groups, respectively (Fig. 4d, e). Alizarin Red S (ARS) staining showed that the density and area of calcium nodules in the MWCNT groups (especially the 1% concentration group) were significantly higher than those in the nHA group and control group, with some nodules showing a tendency to fuse (Fig. 4b). Quantitative detection results were consistent with the ALP activity trends: the 1% MWCNT group had the highest deposition, the 0.5% and 0.2% MWCNT groups were approximately 1.3 times that of the nHA group, and the control group had the lowest deposition (Fig. 4f). The above results collectively indicate that MWCNT scaffolds can significantly enhance the osteogenic differentiation potential of rBMSCs. Scratch assays further revealed that MWCNT scaffolds significantly improved the migration capacity of rBMSCs: the migration rate at 24 h was significantly higher in the 1% group (63.19%), the 0.5% group (52.53%), and the 0.2% group (51.2%) compared to the nHA group (42.79%) and the control group (34.18%) (Fig. 4c, g). Based on the above results, MWCNT scaffolds, through their immunomodulatory effects, can synergistically promote rBMSC migration and osteogenic differentiation (including early ALP activity and late mineralization deposition), providing a favorable microenvironment for bone regeneration.

As a core member of the TGF-β superfamily, BMP-2 significantly promotes bone formation by inducing osteoblast differentiation.^36^ Immunofluorescence staining revealed that the bone morphogenetic protein-2 (BMP-2) protein expression intensity was significantly higher in the MWCNT scaffold group (especially the 1% MWCNT group) than in the nHA group and the control group (Fig. S5a,S5d). After 7 days of co-culture, protein expression analysis further confirmed that, compared with the control group, Runx2 (a key transcription factor regulating osteogenic differentiation) expression was significantly increased in the nHA group but still lower than that in the MWCNT groups (Fig. S5b, S5c). At the same time, BMP-2 and type I collagen (COL-1, key matrix components for osteogenic differentiation and cell adhesion) expression was significantly increased in the MWCNT groups (especially at 1% concentration); expression levels were significantly higher than those in the nHA group and the control group (Fig. S5b, S5c).

Genetically, the MWCNT groups had significantly greater levels of mRNA expression for alkaline phosphatase (ALP, an early osteogenic marker), Runx2, COL-1, and osteopontin (OPN, involved in bone mineralization) compared to the control group (Fig. S5e). Based on the above results, MWCNT scaffolds significantly promoted the expression of osteogenic-related factors (BMP-2, Runx2, COL-1, ALP, OPN) at the protein and gene levels, suggesting their excellent osteogenic differentiation-promoting ability. This effect may be related to the scaffold-mediated polarization of macrophages toward the pro-repair M2 phenotype, which secretes osteogenic factors such as BMP-2, thereby establishing a bone immune regulatory microenvironment conducive to bone regeneration.

### MWCNT bionic scaffolds mediate angiogenesis through macrophage activation

Angiogenesis is a key step in bone healing, as the vascular network it forms provides essential nutrients and signaling molecules for repairing tissue cells. Insufficient angiogenesis can lead to cell death or poor repair tissue integration.^37^ Therefore, the development of functional biomaterials with effective angiogenic activity is crucial for promoting bone healing. This study evaluated the immunomodulatory effects of MWCNT scaffolds on angiogenesis in vitro using scratch assays, in vitro tubulation assays, immunofluorescence staining, and RT-PCR detection. Given the central role of endothelial cell migration in angiogenesis, scratch assays were first performed using human umbilical vein endothelial cells (HUVECs). As shown in Fig. S6a, the scratch area decreased in all groups as the culture time increased. After 24 h of culture, the scratch area in the MWCNT-containing scaffold groups (0.2%, 0.5%, and 1% MWCNT) was significantly smaller than that in the pure nHA group and the blank control group (Control). Quantitative analysis (Fig. S6b) showed that the cell migration rates after 24 h were as follows: 1% MWCNT group (93.44%), 0.5% MWCNT group (74.36%), 0.2% MWCNT group (73.20%), nHA group (64.53%), and control group (44.34%). The migration rates of all MWCNT groups were significantly higher than those of the nHA group and the control group. These results confirm that MWCNT scaffolds can significantly enhance the migration ability of HUVECs.

Further evaluation of the scaffold’s proangiogenic activity was conducted via in vitro tube formation assays. After 4 h of culture, HUVECs in all groups extended protrusions and connected to form vascular-like structures. Compared with the control group, both the MWCNT group and the nHA group significantly enhanced vascular formation. Among them, the 1% MWCNT group had the highest number of vascular crossings and total tube length, followed by the 0.5% and 0.2% MWCNT groups. The results of these three groups were significantly superior to those of the nHA group and the control group (Fig. 4j–l). These data indicate that MWCNT scaffolds can effectively promote endothelial cell migration and in vitro angiogenesis. To investigate the molecular mechanism, we detected the expression of vascular endothelial growth factor (VEGF) protein (immunofluorescence staining) and the expression of matrix metalloproteinase MMP2 and MMP9 genes (RT-PCR). The results were consistent with the trends observed in the previous functional experiments: the MWCNT group, especially the 1% concentration group, significantly upregulated VEGF protein expression (Fig. 4h, i) and the mRNA levels of MMP2 and MMP9 (Fig. 4m). VEGF immunofluorescence staining in HUVECs revealed a visibly stronger VEGF signal in the MWCNT-containing groups compared with the nHA group and the control (Fig. 4h). Semi-quantitative analysis further confirmed that VEGF-positive area fraction was significantly increased in the MWCNT groups, with the 1% MWCNT group showing the highest VEGF expression among all groups (Fig. 4i). These results indicate that the MWCNT-containing scaffolds enhanced the pro-angiogenic phenotype of HUVECs, as reflected by elevated VEGF expression. In summary, the results of this study indicate that MWCNT scaffolds can effectively promote endothelial cell migration, in vitro vascular formation, and the expression of related pro-vascular factors. Their mechanism of action may be related to mediating M2 macrophage polarization and constructing a bone immune regulatory microenvironment conducive to angiogenesis.

### In vivo immunoreactivity of MWCNT bionic scaffolds

Using a rat balloon model to evaluate the in vivo immune response of each group of scaffolds. Hematoxylin and eosin (HE) staining showed that a significantly thickened continuous fibrous capsule layer (466.76 ± 105.49 μm) was observed around the graft in the nHA group 7 days after implantation. In contrast, the thickness of the fibrous capsule layer was significantly reduced in the MWCNT-containing scaffold group (0.5% MWCNT: 209.14 ± 78 μm; 0.2% MWCNT: 201.61 ± 71.75 μm), with the thinnest fibrous layer observed in the 1% MWCNT group (57.00 ± 25.56 μm) (Fig. S7a,S7c). Immunofluorescence double staining results showed that F4/80^+^iNOS^+^ (M1 type) macrophages were significantly more abundant in the fibrous layer of the nHA group than in the 0.5% MWCNT and 0.2% MWCNT groups. Conversely, F4/80^+^CD206^+^ (M2-type) macrophages were more prevalent in the MWCNT scaffold group (Fig. S7b,S7d, S7e). It is worth noting that the 1% MWCNT group exhibited the highest proportion of M2 macrophages and the lowest proportion of M1 macrophages, demonstrating a clear dose-dependent effect. In vivo experiments confirmed that MWCNT scaffolds effectively promote the polarization of macrophages toward the pro-regenerative M2 phenotype, significantly reducing inflammatory responses at the implantation site (as evidenced by thinner fibrous capsules and changes in the M1/M2 ratio), thereby facilitating the integration of the graft with host tissue. These results are consistent with the immunomodulatory effects observed in in vitro studies, collectively indicating that MWCNT scaffolds can promote bone regeneration by regulating the inflammatory microenvironment.

### In vivo evaluation of MWCNT bionic scaffolds modulating bone immunity for repair of bone defects

**Fig. 5:**
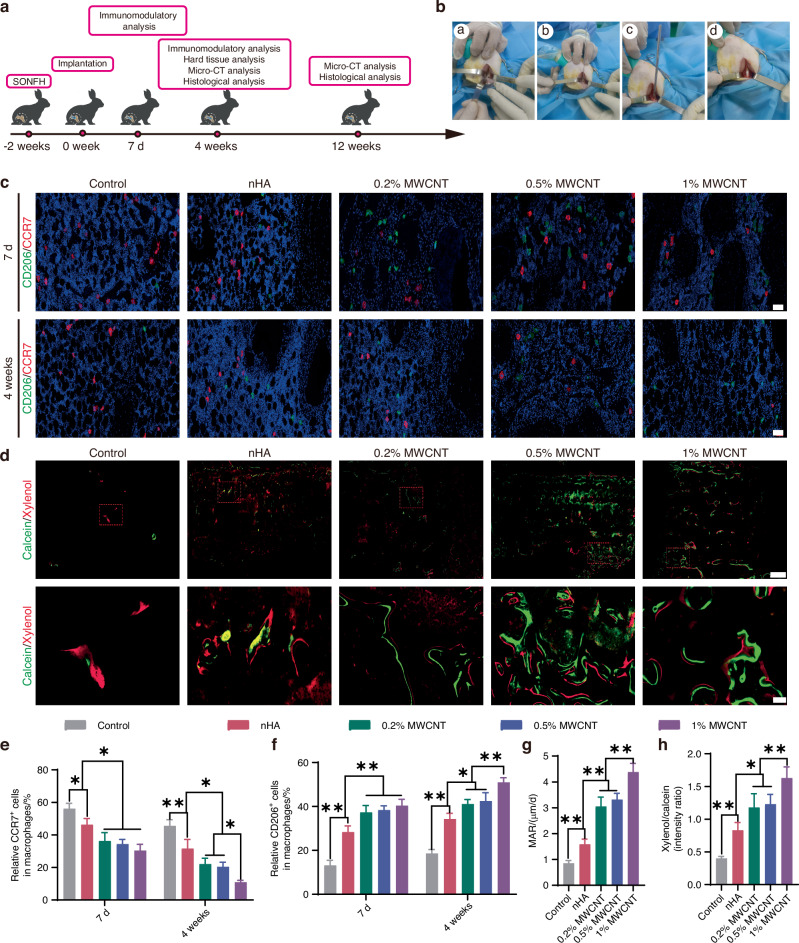
MWCNT bionic scaffolds’ in vivo effects on macrophage polarization in rabbit SONFH. **a** In vivo experiment schedule and setup. **b** Decompression scaffold placement and femoral head drilling surgery. (Panel **a** shows the femur and opening; **b** shows the direction and channel of the femoral neck probed by a Kirschner needle; **c** shows the orthopedic drill drilling; and **d** shows the implantation of material into the drilled channel.). **c** After 7 days and 4 weeks of implantation, the sample rabbit femoral heads were immunofluorescently stained with CD206 and CCR7 (CCR7 was labeled red, CD206 was stained green, and nuclei were stained blue). There is a 100 μm scale bar. **d** Four-week post-implantation fluorescence microscopy picture of calcein/xylenol orange. Using a fluorescent microscope, the sections were observed to exhibit xylenol orange at 580 nm excitation and calcein green at 470 nm excitation, with xylenol orange exhibiting red fluorescence and calcein green fluorescence. 1 000 μm and 100 μm, respectively, made up the scale bar. **e**, **f** Quantitative evaluation of immunofluorescence staining following implantation for seven and four weeks. **g** Four weeks following implantation, a quantitative evaluation of the mineralization deposition rate (MAR) in the defect area. **h** Bone reconstruction rate four weeks post-implantation (shown by the ratio of calcium to xylenol). *P < 0.05; **P < 0.01; ***P < 0.001

Four weeks after the establishment of the steroid-induced osteonecrosis (SONFH) model, HE staining of femoral head tissue showed (Fig. S8): Compared with the normal group, the SONFH group exhibited sparse trabecular bone structure, disordered arrangement, and significantly increased bone lacuna vacancy rate (63.75% ± 8.73% vs. the normal group), with extensive infiltration of fat cells.^38^ These pathological features confirm the successful establishment of the SONFH rabbit model. After successful modeling, scaffolds were implanted into the femoral heads of rabbits in each group (surgical schematic diagram shown in Fig. 5b), and tissue samples were collected and analyzed at 7 days, 4 weeks, and 12 weeks post-surgery (Fig. 5a). To validate the MWCNT-induced M2 polarization of macrophages observed in vitro, postoperative tissue sections were stained for CCR7 (M1 marker) and CD206 (M2 marker). Immunofluorescence staining results showed that, compared with the nHA group and the control group, the MWCNT scaffold implantation group had a significantly higher proportion of CD206^+^ M2 macrophages and a significantly lower proportion of CCR7^+^ M1 macrophages. This effect was concentration-dependent, with the highest M2 ratio and lowest M1 ratio observed in the 1% MWCNT group (Fig. 5c, e, f). The above data confirm that MWCNT scaffolds can effectively promote the conversion of local macrophages from the pro-inflammatory M1 phenotype to the reparative M2 phenotype, thereby alleviating inflammatory responses in the pathological environment of SONFH and creating a favorable bone immune microenvironment for subsequent bone regeneration.

Double fluorochrome labeling (xylenol orange and calcein) was performed to evaluate dynamic mineralization in vivo. Xylenol orange and calcein were administered at 10 days and 3 days prior to euthanasia, respectively, to label two mineralization fronts. The mineral apposition rate (MAR) was calculated as the mean inter-label distance measured between the two fluorescent labels divided by the inter-label interval (μm/d), following standardized bone histomorphometry nomenclature. Because fluorescence intensity can be affected by imaging settings and section thickness, we did not interpret fluorescence intensity as mineral density; instead, the spacing between labels (MAR) was used as the quantitative index of mineralization dynamics.^39–41^ Through calcein and xylenol orange fluorescence labeling, the dynamics of new bone formation and mineralization were assessed. Fluorescence microscopy imaging showed that the amount of new bone formation and the degree of mineralization in all MWCNT groups were significantly higher than those in the nHA group and the control group, with the 1% MWCNT group showing the best results (Fig. 5d). At 4 weeks post-surgery, the xylenol orange/calcein fluorescent intensity ratio in the MWCNT group (especially at 1% concentration) was significantly higher than that in the nHA group and control group (Fig. 5h), indicating enhanced bone formation rate, bone remodeling rate, and bone turnover activity. Quantitative analysis of the mineral apposition rate (MAR, μm/day) further confirmed that the mineral apposition rate in the defect area was significantly higher in the MWCNT group (especially at 1% concentration) than in the control group (Fig. 5g). In summary, MWCNT scaffold implantation significantly promoted new bone formation, mineral deposition, and bone remodeling in the SONFH defect area, accelerating bone tissue repair.

### New bone formation analyzed by Micro CT

**Fig. 6:**
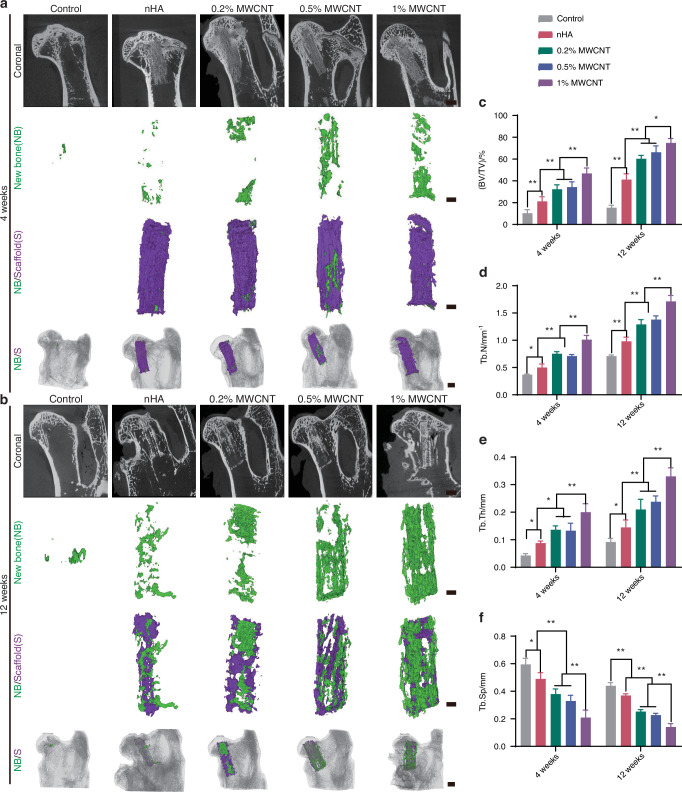
In vivo micro-CT analysis of new bone growth in rabbit femoral head lesions. **a**, **b** Micro CT imaging of the femoral head’s 2D coronal view and 3D reconstruction at 4 and 12 weeks (**a**, **b**) following scaffold implantation in each group (green and purple, respectively). The scale bars are arranged as follows: 3 mm, 1 mm, 1 mm, and 3 mm. **c**–**f** Analysis of bone volume fraction (BV/TV) and bone trabecular parameters (Tb.N, Tb.Th, and Tb.Sp) in the target region in each group following scaffold implantation for 4 and 12 weeks. *P < 0.05; **P < 0.01; ***P < 0.001

The osteogenic effect of MWCNT scaffolds was evaluated using a rabbit model of femoral head defects. According to a micro-CT study, four weeks following surgery, the control group had relatively little new bone formation and clear bone defect margins; the nHA group had some new bone formation, but the defect was still visible; and the MWCNT group had significantly more new bone formation, particularly at 1% concentration (Fig. 6a). At 12 weeks following surgery, the MWCNT group showed more significant bone filling, but the defects in the control and nHA groups were not completely healed (Fig. 6b). Quantitative analysis verified (at 4 and 12 weeks post-surgery) that the MWCNT group had significantly lower trabecular separation (Tb.Sp), a concentration-dependent relationship (the 1% group being optimal), and significantly higher bone volume fraction (BV/TV), trabecular number (Tb.N), and trabecular thickness (Tb.Th) than the control and nHA groups (Fig. 6c–f).

According to micro-CT analysis, the MWCNT-containing scaffold groups exhibited enhanced new mineralized tissue formation within the femoral head defect in the SONFH rabbit model, as indicated by increased BV/TV, Tb.N, and Tb.Th together with decreased Tb.Sp compared with the control and nHA scaffold groups. Here, ‘new bone’ refers to mineralized tissue formed within the predefined cylindrical defect VOI after implantation, quantified by micro-CT segmentation excluding the radiopaque scaffold phase (thresholding + scaffold masking/subtraction; see Methods). These micro-CT findings indicate improved bone regeneration within the defect region in the MWCNT-containing scaffold groups.

### New bone formation by histologic analysis

**Fig. 7:**
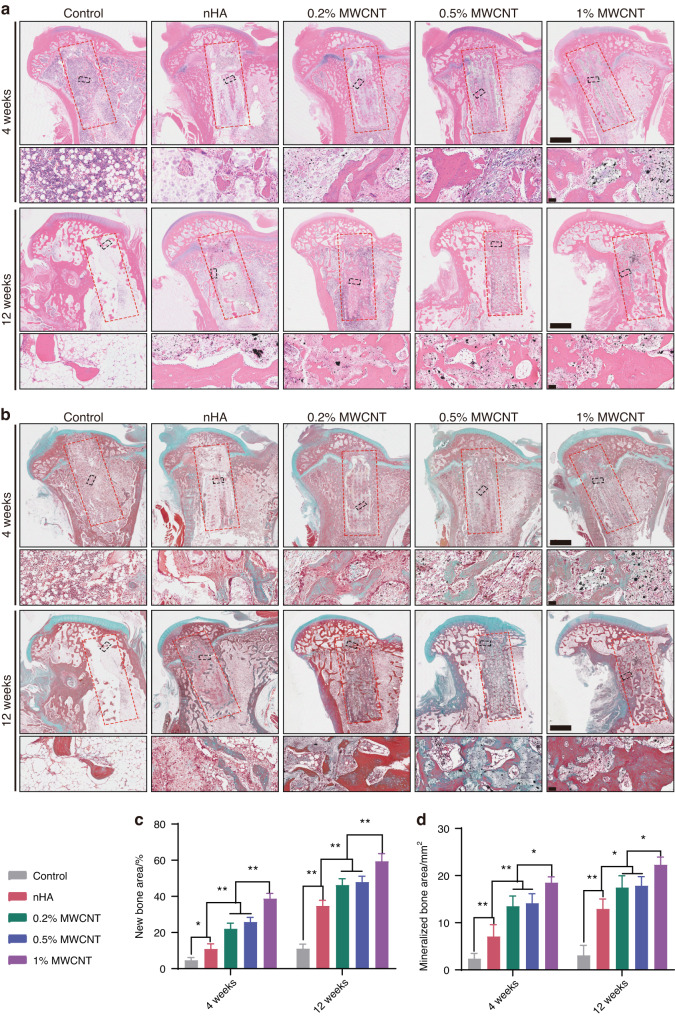
In vivo histological staining examination of the bone defect area 4 and 12 weeks following implantation of MWCNT scaffolds of various compositions, including HE and Goldner staining. **a** HE staining of bone tissues at 4 and 12 weeks post-scaffold implantation; the black box shows the high magnification (100X) image of the bone defect area, and the red box shows the bone defect area itself. The scales measured 2 mm and 100 μm. **b** Bone tissue Goldner staining at 4 and 12 weeks following scaffold implantation. Black boxes show the locations of bone flaws magnified at high magnification (100X), while red boxes show the areas of bone defects. The scales measured 2 mm and 100 μm. **c** Quantitative evaluation of the percentage of newly formed bone tissue at 4 and 12 weeks postoperatively. **d** Quantitative study of the percentage of mineralized bone area at 4 and 12 weeks after surgery. *P < 0.05; **P < 0.01; ***P < 0.001

The scaffold’s ability to promote bone regeneration was further evaluated through histological analysis using Goldner trichrome staining and H&E staining. In line with micro-CT findings, H&E staining (Fig. 7a, c) revealed that the defect area in the control group was mostly composed of fibrous tissue (bridging the borders of the host bone) at 4 and 12 weeks after surgery. This group also had a low capability for bone regeneration. On the other hand, the MWCNT group demonstrated a notable rise in new bone tissue in the defect area, particularly at the 1% concentration. The greatest material-bone integration and bone regeneration results were shown by the 1% MWCNT group in particular. At 4 and 12 weeks after implantation, further Goldner trichrome staining was carried out to evaluate bone mineralization and bone maturity (Fig. 7b, d). According to Goldner triple staining, the MWCNT group significantly increased the area of mature mineralized bone tissue (green) and osteoid (orange/red) when compared to the control group and nHA group. This suggests that the MWCNT group efficiently promotes bone matrix mineralization and remodeling. The above findings undeniably demonstrate that MWCNT scaffolds exhibit exceptional bone repair ability in SONFH bone defect sites by greatly enhancing new bone formation, mineralization maturation, and material-bone integration capacity. Simultaneously, it was noted that the scaffolds gradually deteriorated as new bone grew into them. This suggests that the scaffolds’ degradation process and new bone creation demonstrated a synergistic dynamic alternation, which may help to enhance bone regeneration.

**Fig. 8:**
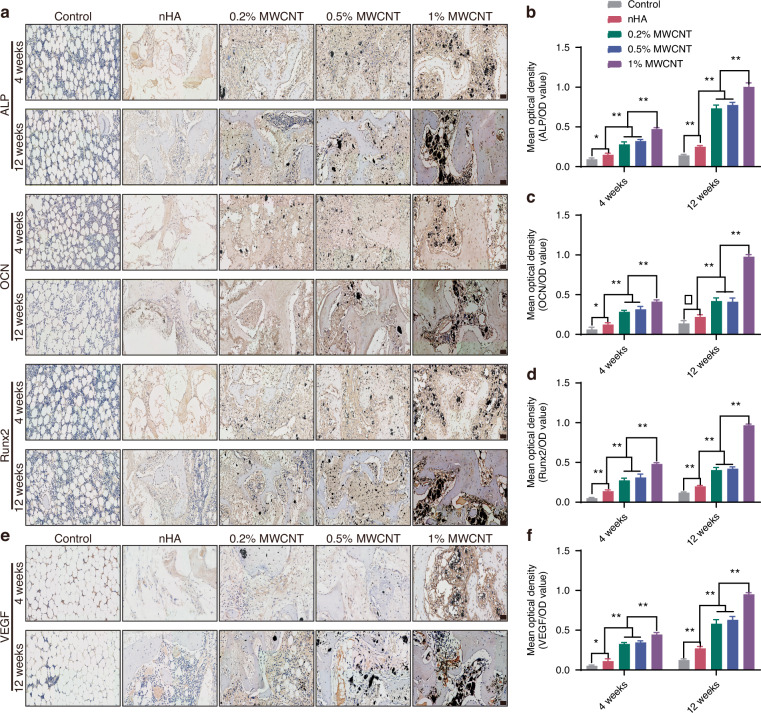
In vivo repair of bone defects after scaffold implantation was assessed by histologic and immunofluorescent staining. **a**, **e** Immunohistochemical staining showing the expression of ALP, OCN, Runx2, and VEGF in the bone defect area 4 and 12 weeks after scaffold implantation. The scale bar was 50 μm. **b**–**d, f** Positive areas for ALP, OCN, Runx2, and VEGF (**f**) expression were quantified using ImageJ software. *P < 0.05; **P < 0.01; ***P < 0.001

To evaluate the efficacy of implanted scaffolds in promoting osteogenesis and biomineralization during bone repair, immunohistochemical staining analysis of key osteogenic markers Runx2, OCN, and ALP was performed at 4 and 12 weeks post-surgery. Runx2 is a core transcription factor regulating osteoblast differentiation, capable of activating the expression of bone formation-related genes, including ALP and OCN; OCN, as an osteoblast-secreted protein, is a key factor in bone matrix mineralization and a specific marker of mature bone tissue. Immunohistochemical analysis results showed (Fig. 8a–d) that the expression levels of ALP, OCN, and Runx2 in the MWCNT group were significantly higher than those in the control group and nHA group. Notably, the 1% MWCNT group exhibited the highest intensity of positive staining for the markers, with expression levels decreasing progressively in the 0.5% and 0.2% MWCNT groups, indicating that the efficacy of MWCNT scaffolds in promoting bone matrix maturation and mineralization is concentration-dependent, thereby confirming their superior bone regeneration capacity. In addition, Fig. S10 shows that, compared with the control group, no significant pathological changes were detected in the major organs (heart, liver, spleen, and kidney) of all experimental groups, confirming that the scaffold system has good in vivo biocompatibility.

Angiogenesis is closely coupled with osteogenesis, in which angiogenesis not only provides essential blood supply and nutritional support for new bone formation,^42,43^ but factors secreted by endothelial cells (such as VEGF) can also directly promote the osteogenic differentiation of BMSCs.^42^ As one of the most effective angiogenesis factors, the expression level of VEGF in the MWCNT scaffold group (assessed by immunohistochemical staining and quantitative analysis) was significantly higher than that in the control group and nHA group (Fig. 8e, f). To assess the effect of scaffolds on neovascularization, the vascular endothelial marker CD31 was detected by immunofluorescence at 4 and 12 weeks postoperatively.^44^ The results showed that the MWCNT group had the highest CD31 expression level, significantly higher than the nHA group and the control group (Fig. S9a, b), indicating that the scaffold can effectively promote angiogenesis and maturation. VEGF staining revealed enhanced VEGF expression in the newly formed tissue of the MWCNT scaffold groups compared with the Control and nHA groups (Fig. 8e, f). Consistently, CD31 staining showed increased CD31⁺ microvessel density in the defect region in the MWCNT groups, with the 1% MWCNT group exhibiting the most pronounced vascular ingrowth (Fig. S9a, b). Comprehensive immunohistochemistry (VEGF), immunofluorescence (CD31), and in vitro experimental results all consistently confirmed that the MWCNT scaffold has significant angiogenic capacity. The enhanced vascularization improves local blood flow, accelerates material exchange in necrotic areas, and promotes bone formation and repair through paracrine osteogenic factors.^34,45^ In conclusion, MWCNT scaffolds facilitate the polarization of local macrophages from pro-inflammatory M1 type to reparative M2 type. This results in the secretion of pro-osteogenic/pro-angiogenic and anti-inflammatory factors (like VEGF), the recruitment of endogenous MSCs, and the induction of angiogenesis. These actions convert the inflammatory microenvironment into a reparative microenvironment, which ultimately leads to the successful repair of osteonecrosis.

### Underlying molecular mechanisms of MWCNT regulation of M2 macrophage polarization

**Fig. 9:**
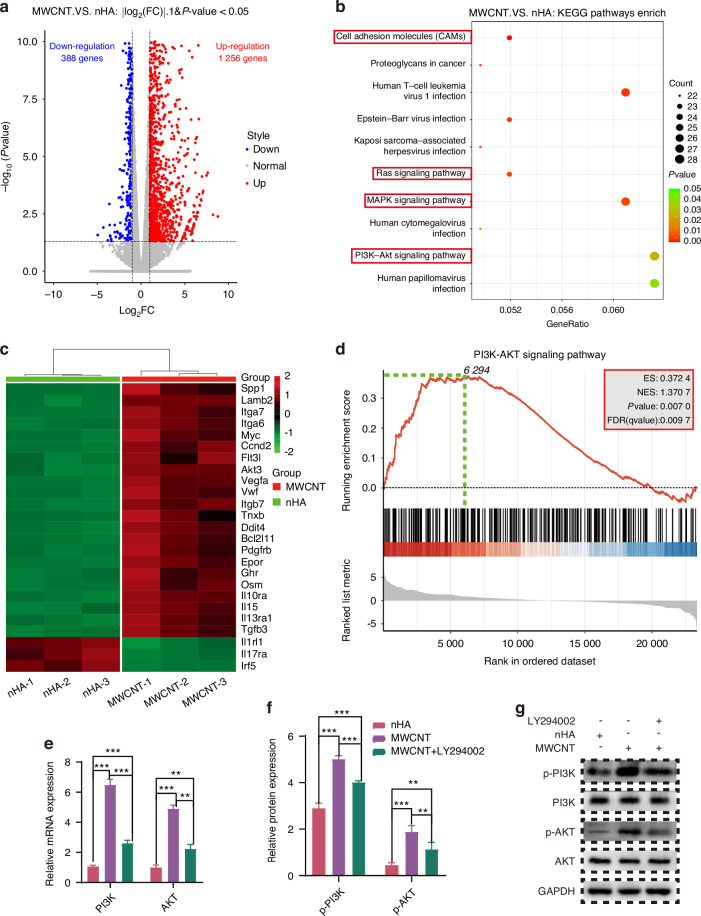
Mechanistic analysis of macrophage polarization mediated by MWCNT. **a** Volcano map of the genes that were expressed differently in the MWCNT and nHA groups. (Genes that are up-regulated are red; genes that are down-regulated are green). **b** KEGG pathway enrichment analysis for genes that are expressed differently in MWCNT than in nHA. **c** Heatmap analysis of M1 and M2 phenotype-related variables and differentially expressed genes in the PI3K-AKT signaling pathway in MWCNT versus nHA. **d** Gene sets involved in the PI3K-AKT signaling pathway, as shown on a GSEA enrichment map. The enrichment score of the PI3K/AKT pathway gene sets in MWCNT increased significantly, according to GSEA. **e** RT-PCR test demonstrating PI3K and AKT gene levels in the PI3K/AKT signaling cascade. **f** Statistical study of p-AKT and p-PI3K is semi-quantitative. **g** Western blotting assay displaying PI3K, p-PI3K, AKT, and p-AKT protein levels. *P < 0.05; **P < 0.01; ***P < 0.001

To elucidate the immunoregulatory mechanism of MWCNT scaffold-mediated macrophage polarization, transcriptomic sequencing (RNA-Seq, triplicates) was performed on RAW 264.7 cells cultured on MWCNT scaffolds. The results showed that there were 19 638 genes expressed in both groups, while the MWCNT group and nHA group had 2 120 and 1 715 differentially expressed genes, respectively (Fig. S11a). Volcano plot analysis (Fig. 9a) revealed that, compared to the nHA group, the MWCNT group had significantly differentially expressed genes (DEGs), including 1 256 upregulated and 388 downregulated genes. Using this DEGs dataset, we conducted a three-level Gene Ontology (GO) functional enrichment analysis: cellular component, biological process, and molecular function (Fig. S11b). Actin cytoskeleton organization, positive regulation of programmed cell death, positive regulation of immunological response, inflammatory response, regulation of cell adhesion, and regulation of cellular response to stress were among the top 30 enriched phrases in the biological process category. Macrophage polarization traits are closely associated with these enriched pathways. MWCNT scaffolds’ mechanism of controlling macrophage polarization was molecularly supported by transcriptome analysis, which showed that they drastically altered the gene expression profiles of macrophages and enriched pathways controlling immune responses, inflammation, and cell behavior.

To further investigate the potential signal transduction mechanisms, we performed Kyoto Encyclopedia of Genes and Genomes (KEGG) pathway enrichment analysis on differentially expressed genes. The results showed that, compared to nHA scaffolds, MWCNT scaffolds were significantly enriched in the PI3K-Akt, MAPK, cell adhesion molecules (CAMs), and Ras signaling pathways (Fig. 9b). Notably, the PI3K-Akt signaling pathway has been previously shown to be a key pathway regulating the polarization of macrophages toward the M2 phenotype.^46–49^ Its activation is a necessary condition for promoting M2 polarization,^47–49^ while inhibition of this pathway weakens M2 polarization and may enhance M1 polarization.^48^ Based on this, we further analyzed the expression profiles of key genes in the PI3K-AKT pathway and factors related to the M1/M2 phenotype. Heatmap analysis (Fig. 9c) showed that the expression of PI3K-AKT pathway-related genes (such as Spp1, Lamb2, Itga6, Itga7, Itgb7, and Myc) was significantly upregulated in the MWCNT group. At the same time, pro-inflammatory M1 phenotype-related factors (IL17ra, IL1rl1, and IRF5) were downregulated, while reparative M2 phenotype-related factors (IL10ra, IL15, IL13ra1, and TGFβ3) were upregulated. This result was independently validated by gene set enrichment analysis (GSEA): GSEA showed that the PI3K-AKT pathway-characteristic gene set was significantly upregulated and highly enriched in the MWCNT group (Fig. 9d). In summary, transcriptomic data consistently indicate that MWCNT scaffolds mediate macrophage polarization toward the M2 phenotype by significantly activating the PI3K-AKT signaling pathway and regulating the expression of M1/M2-related factors.

To validate the key role of PI3K-AKT signaling pathway activation in scaffold-mediated macrophage polarization, we used LY294002, a reversible inhibitor that can penetrate cells and specifically target the PI3K ATP binding site.^50^ Gene expression analysis revealed that the mRNA levels of PI3K and AKT were significantly higher in the MWCNT scaffold group compared to other control groups (Fig. 9e). Western blot results further confirmed that the MWCNT scaffold significantly promoted the phosphorylation of PI3K and AKT proteins (Figs. 9f, g). Notably, treatment with LY294002 partially reversed the promoting effect of MWCNT scaffolds on PI3K/AKT phosphorylation. These results collectively confirm that MWCNT scaffolds drive macrophage polarization toward the M2 phenotype by activating the PI3K-AKT signaling pathway.

## Discussion

In this study, we developed a coral-inspired hierarchical porous biomimetic scaffold integrating multi-walled carbon nanotubes (MWCNTs) with nano-hydroxyapatite (nHA) to address the “immune freeze” microenvironment in steroid-induced osteonecrosis of the femoral head (SONFH). Our key findings demonstrate that the MWCNT scaffold effectively reprograms macrophages from the pro-inflammatory M1 to the reparative M2 phenotype via PI3K-AKT pathway activation, thereby enhancing osteogenesis, angiogenesis, and bone regeneration in both in vitro and in vivo models, offering a novel strategy to overcome the limitations of current SONFH treatments.

The fabrication and characterization results reveal that the MWCNT-integrated scaffolds maintain a biomimetic hierarchical porous structure akin to trabecular bone, with uniform elemental distribution and mechanical properties suitable for cancellous bone support. Here, the term ‘coral-inspired’ does not indicate an identical coral-like appearance, but rather refers to a hierarchical porous organization analogous to natural coral skeletons featuring interconnected macroporous channels and abundant micro-/nano-scale porosity. In our design, the orthogonal 0°–90° lattice provides uniform macropores (~400 μm) for nutrient/oxygen transport, while the freeze-drying process and the nHA/MWCNT composite within the struts contribute additional micro-/nano-porosity and increased surface area, thereby facilitating cell attachment and tissue ingrowth. Such a hierarchical pore system is functionally relevant for bone defect repair, as macropores facilitate perfusion and tissue infiltration, whereas micro-/nano-porosity enhances surface area and adhesion sites, supporting coordinated angiogenesis and osteogenesis during healing. This structure facilitates cell infiltration and nutrient transport, as evidenced by the interconnected macropores ( ~ 400 μm) and micropores (~9 μm), which align with optimal pore sizes for bone tissue engineering.^32,33^ The sustained release of MWCNTs and controlled degradation further support long-term bioactivity, enabling gradual scaffold resorption synchronized with new bone formation. Biocompatibility assessments indicate that MWCNTs enhance cell spreading, proliferation, and cytoskeletal reorganization in RAW 264.7 macrophages and rat bone marrow mesenchymal stem cells (rBMSCs), likely due to the nanotopological cues and high specific surface area of MWCNTs, which mimic extracellular matrix features and promote focal adhesion formation.^51^ These observations underscore the scaffold’s role in creating a conducive microenvironment for cellular responses critical to bone repair.

Immunomodulation experiments highlight the scaffold’s capacity to reverse glucocorticoid-induced M1 macrophage polarization, promoting M2 conversion and altering cytokine profiles to favor anti-inflammatory and pro-regenerative factors such as IL-10, BMP-2, and VEGF. This shift is mechanistically linked to PI3K-AKT pathway activation, as confirmed by transcriptomic analysis showing upregulation of pathway-related genes and downregulation of M1 markers. Such reprogramming mitigates the “immune freeze” in SONFH by reducing pro-inflammatory cytokines like TNF-α, which impair osteoblast function, and enhancing osteogenic signals that drive rBMSC differentiation.^52^ In co-culture systems, this immunomodulation translated to elevated ALP activity, calcium nodule formation, and migration in rBMSCs, with corresponding increases in osteogenic markers like Runx2, COL-1, and OPN. Similarly, angiogenesis assays demonstrated enhanced endothelial cell migration, tube formation, and expression of VEGF, MMP2, and MMP9, illustrating how M2 macrophages orchestrate vascular-osteogenic coupling essential for SONFH repair.^53^ In vivo, the scaffold reduced fibrous capsule thickness and promoted M2 dominance in a rat air pouch model, while in the SONFH rabbit model, it accelerated new bone formation, with enhanced mineralized tissue formation, and vascularization, as quantified by micro-CT parameters (e.g., increased BV/TV and Tb.N) and histological markers (e.g., elevated Runx2, OCN, ALP, and CD31). These integrated effects suggest that the scaffold actively remodels the pathological niche, synchronizing immune regulation with tissue regeneration. To enhance interpretability and methodological transparency, we further specify the definition and analytical scope of the micro-CT–derived “new bone” metrics as follows.

To interpret the micro-CT findings appropriately, it should be emphasized that micro-CT in this study provides quantitative evidence of structural regeneration within the predefined defect volume rather than mechanical fixation. Accordingly, we avoid the term ‘osseointegration’, as mechanical integration tests were not performed. Here, ‘new bone’ refers to mineralized tissue formed within the cylindrical defect VOI after implantation, quantified by threshold-based segmentation with exclusion of the radiopaque scaffold phase (mask subtraction), and the reported indices (BV/TV, Tb.N, Tb.Th, and Tb.Sp) should be interpreted as changes in trabecular micro-architecture within that VOI. In addition, woven-versus-lamellar bone composition and remodeling-specific indices were not quantified (e.g., no polarized-light quantification), and local calibrated mineral density/TMD (g/cm³) was not separately measured; therefore, we refrain from using the term ‘bone maturation’ and restrict conclusions to the measured structural parameters. Collectively, these findings suggest that the MWCNT-containing scaffolds may contribute to improved bone repair in the SONFH model; however, this outcome should be interpreted cautiously as a multifactorial process potentially involving scaffold osteoconductivity/architecture, mechanical stabilization, and the local immune milieu rather than immune modulation alone.^54,55^ Future work will further validate scaffold–bone integration and tissue-level maturation by incorporating mechanical integration tests (e.g., push-out/pull-out), quantitative histological assessment of woven versus lamellar bone, and calibrated mineral density mapping.

Comparisons with existing literature underscore the innovation of our approach. Traditional biomaterials like PLGA/TCP scaffolds promote osteogenesis but lack immune modulation, failing to address GC-induced M1 stasis.^56^ Similarly, PEEK-based materials require surface modifications for bioactivity, yet overlook microenvironmental reprogramming.^57^ Our MWCNT scaffold advances beyond these by integrating immunomodulatory properties, aligning with studies on carbon nanomaterials that enhance M2 polarization via nanotopography.^16,58^ For instance, while black phosphorus scaffolds activate PI3K-AKT for osteodifferentiation,^10^ they do not target SONFH-specific immune imbalances; our work fills this gap by demonstrating pathway-specific reversal of “immune freeze.” In contrast to HA-only scaffolds, which exhibit slow degradation and limited immune interaction,^11^ our composite achieves tunable mechanics and sustained MWCNT release, consistent with findings on CNT-reinforced ceramics improving toughness without cytotoxicity.^59^ Angiogenic outcomes echo reports on VEGF-loaded nanomaterials,^60^ but our scaffold’s endogenous promotion via M2-secreted factors offers superior integration, differing from exogenous delivery systems prone to burst release.^61^ In vivo bone regeneration surpasses core decompression alone, which alleviates pressure but sustains inflammation,^62^ and outperforms allogeneic grafts limited by rejection. Innovations include the coral-inspired hierarchy, enabling efficient nutrient transport absent in uniform porous scaffolds^63^ and concentration-dependent effects, building on dose-response studies in immunomodulatory nanomaterials.^64^ Differences from prior works, such as higher M2 induction than graphene oxide scaffolds,^65^ may stem from MWCNTs’ unique conductivity and surface energy, enhancing pathway activation. This positions our scaffold as a bridge between passive osteoconductive materials and active immunoregulatory platforms, addressing knowledge gaps in SONFH pathophysiology.^66^

The significance of these findings extends to advancing theoretical understanding of immune-osteogenic interactions in pathological bone environments, providing mechanistic insights into PI3K-AKT-mediated macrophage plasticity that could inform broader regenerative strategies. Clinically, the scaffold offers a promising adjunct to core decompression, potentially reducing the progression to joint replacement in SONFH patients by enhancing postoperative bone formation and vascularization. Its biocompatibility and degradability suggest translational potential for personalized implants via 3D printing, impacting orthopedic interventions and reducing global medical burdens affecting millions annually. Future applications may include drug loading for sustained release, expanding to other immune-mediated bone disorders like osteoarthritis or fracture non-unions.

Despite these strengths, limitations exist. The rabbit SONFH model, while replicating key pathological features, may not fully capture human disease heterogeneity, potentially limiting generalizability; larger animal models or clinical trials could validate efficacy across species.^38^ Sample sizes in in vivo experiments, though statistically powered, were modest, and long-term degradation products’ effects warrant further toxicology studies to ensure safety. The focus on PI3K-AKT overlooks potential crosstalk with other pathways like MAPK, which could be explored using multi-omics approaches. To mitigate these, future work should incorporate human-derived cells and extended follow-up periods, enhancing translational relevance without undermining the current evidence of scaffold efficacy.

In conclusion, this study establishes MWCNT scaffolds as an effective platform for immune reprogramming in SONFH, driving M2 polarization and coupled osteogenesis-angiogenesis to promote repair. Future directions include optimizing MWCNT concentrations for human applications and integrating bioactive molecules to target additional pathways, paving the way for advanced regenerative therapies.

## Materials and methods

### Materials

Multiwalled carbon nanotubes (MWCNT) were purchased from Chengdu Organic Chemistry Co., Ltd. of the Chinese Academy of Sciences, and hydroxyapatite nanoparticles (nHA) and poly(lactic acid)-glycolic acid copolymers (PLGA) were purchased from Jiangsu Xianfeng Nano-materials Technology Co. Lipopolysaccharide (LPS), 1,4-dioxane, methyl methacrylate, Xylenol Orange tetrasodium salt, and Calcein were purchased from Sigma-Aldrich Trading Co., Ltd. (Shanghai, China). Bone marrow stem cells (BMSCs) were extracted from rat backbone bone marrow, and P3 to P5 generation BMSCs were used for in vitro cell experiments. Macrophages (RAW 264.7 cells) as well as human umbilical vein endothelial cells (HUVECs) were provided by the Institute of Biochemistry and Cell Biology, Chinese Academy of Sciences (Shanghai, China). Cell culture reagents were purchased from Gibco Life Technologies (USA). Various antibodies, cell detection kits, and ELISA kits were purchased and obtained from Abcam (USA), Cell Signaling Technologies (USA), Life Technologies (USA), Bioss Biosciences (Beijing), Beyotime Biotechnology (Shanghai), and Xinbosheng Biotechnology (Shenzhen). All animal experiments were performed in accordance with the guidelines approved by the Animal Care and Experimentation Committee of West China Hospital of Sichuan University. Guidelines approved by the Animal Care and Experimentation Committee of West China Hospital of Sichuan University were followed.

### Preparation and characterisation of MWCNT bionic scaffolds

To prepare MWCNT bionic scaffolds, dissolve 2.0 g of PLGA in 10 mL of 1,4-dioxane, heated under closed conditions at 60°C with stirring overnight, then 1 g of hydroxyapatite and different concentrations of multi-walled carbon nanotubes (0.02 g, 0.05 g, 0.1 g, and 0 g) were added, stirred for 2 h, and sonicated for 20 minutes to prepare homogeneous printing inks containing 0.2 wt%, 0.5 wt%, and 1 wt% (MWCNT with w/w ratios of 1:50, 1:20, and 1:10) and pure nHA. The plastic syringes containing the above printing inks were then installed on a low-temperature deposition 3D printer (SUNP ALPHA-BP31, Beijing), and the printing parameters were set as follows: Material barrel: 5 mm syringe; nozzle: 400 μm diameter; printing speed is 5 mm/s; extrusion speed is 0.25 mm³/s. Then, at a low temperature of –25 °C, following the pre-designed print path (layer height 200 μm; fiber spacing 400 μm; laying pattern 0°–90°), the printed fibers are stacked layer by layer on a low-temperature receiving plate. Importantly, all scaffold groups were printed using the same CAD model and identical printing parameters, resulting in the same orthogonal lattice macro-architecture across groups; the only variable among groups was the MWCNT content in the composite ink (0%, 0.2 wt%, 0.5 wt%, and 1 wt%). Print the scaffold as cylinders with a diameter of 10 mm (height 3 mm) and 3.5 mm (height 10 mm). The 10 mm diameter (height 3 mm) cylinders are used for in vitro experiments, while the 3.5 mm diameter (height 10 mm) cylinders are used for in vivo experiments. Place the printed scaffold blanks in a –80 °C freezer for further freezing. After 12 h, place the scaffold blanks in a freeze-dryer (–70°C) for 24–48 h to remove the solvent. Prior to cell culture or in vivo experiments, sterilize the MWCNT and nHA scaffolds using ethylene oxide.

Surface morphology and high-resolution microstructure of different scaffolds were observed using a field emission scanning electron microscope (SEM, Thermo Fisher Scientific, USA). Additionally, the elemental distribution and quantitative analysis of the low-temperature deposited printed scaffolds were analyzed using the energy-dispersive X-ray spectrometer (EDX) equipped with the SEM. After grinding the 3D-printed scaffolds into powder, the phase composition and structure of MWCNT or nHA after 3D printing were analyzed using an X-ray diffractometer (XRD, PANalytical B.V., Netherlands). The mechanical properties of the 3D-printed scaffolds, including compressive stress and compressive Young’s modulus, were determined using an electronic universal testing machine (AG, Instron, UK). The compressive Young’s modulus was calculated based on the slope of the stress-strain curve in the linear region. The porosity, pore size, pore volume, pore size distribution, total pore volume, total pore surface area, and median pore size of the 3D-printed scaffolds were determined using a high-performance fully automatic mercury intrusion porosimeter (MIP, Micromeritics, USA).

In vitro degradation experiment: Measure and record the mass of the scaffold material (Ma) and place it in a well plate. Add PBS to each well, then place the plate in an incubator and seal it. On days 1, 4, 7, 14, 21, and 28, remove the scaffold samples from each group, wash and dry them, then measure their dry weight (Mb). The degradation rate is calculated using the formula: Degradation rate = (Ma–Mb)/Ma × 100%. Record the degradation rate, and take the average of three results as the experimental result. Additionally, degradation solutions are collected at each time point, and the absorbance is measured at 800 nm using a U-2000 spectrophotometer to determine the concentration of MWCNTs in the degradation solution. The calibration curve ranges from 0.1 to 20 μg/L MWCNT. Three samples are tested for each scaffold.

### Cytotoxicity and biocompatibility of MWCNT bionic scaffolds of different compositions

To assess the cytotoxicity of MWCNT scaffolds with different compositions produced by 3D printing, live-dead staining was used for evaluation. In brief, after 4 days of culture, BMSCs and RAW264.7 cells were collected by centrifugation (1 500 r/min, 5 min), the supernatant was discarded, the cells were washed with 1× Assay Buffer, resuspended, counted, and adjusted to a cell suspension of 1 × 10⁵–1 × 10⁶ cells/mL; Add 100 µL of staining working solution to a certain amount of cell suspension, then incubate at 37 °C for 15 minutes; Use a 490 ± 10 nm excitation filter to simultaneously detect dead cells (red fluorescence) and live cells (yellow-green fluorescence). Use the Cell Counting Kit-8 (CCK-8, Yeasen Company, Shanghai) to detect and assess the proliferation activity of rBMSCs and RAW 264.7 cells. The method is as follows: Seed 1 × 10⁵ BMSCs and 1 × 10⁵ RAW 264.7 cell suspensions into a 96-well plate and incubate in an incubator for 1 day and 4 days. After incubation, add CCK-8 working solution (10 μL/well) to each well, then place the plate in an incubator for another 3–4 h, and measure the absorbance at 450 nm (microplate reader, Thermo, USA). To observe the adhesion, spreading, and distribution of rBMSCs, 1 × 10⁵ BMSCs were seeded onto 3D-printed scaffolds in a 12-well plate. After 2 days of culture, the cells were fixed and examined by scanning electron microscopy and immunofluorescence staining according to standard procedures.

SEM observation: After culturing for 2 days, the scaffold was fixed with 2.5% glutaraldehyde +2% polyformaldehyde at 4 °C overnight; after washing and dehydration with graded ethanol, it was dried; the sample was then sputter-coated with gold and finally observed under the microscope. Laser confocal microscopy (CLSM, Nikon Instruments, Japan): After culturing for 2 days, wash the scaffold three times with PBS; fix at room temperature for 15 minutes using an immunofluorescence fixation solution; wash the scaffold three times with 0.1% Triton X-100; dilute the Actin (Beyotime Bio, Shanghai) solution with the appropriate ratio of fluorescently labeled secondary antibody diluent; add the staining solution to the slides at the appropriate ratio and incubate at room temperature in the dark for 60 minutes; finally, after washing and DAPI incubation, observe the cell spreading using laser confocal microscopy (CLSM) and take photographs.

### Immunomodulation of macrophage phenotypic transformation under glucocorticoid action by MWCNT bionic scaffolds of different compositions

Following a 24-h pretreatment, the interferon-γ + lipopolysaccharide + dexamethasone-induced RAW264.7 cells were utilized as macrophages in in vitro cell studies. The mass concentrations of lipopolysaccharide, interferon-γ, and dexamethasone were 100 ng/mL, 100 ng/mL, and 10 μmol/L, respectively. The pretreated RAW264. 7 cells were diluted to a concentration of 1 × 10^7^ cells/mL, seeded onto a 24-well plate with embedded slides, and cultured for 24 h, then treated with MWCNT porous scaffolds of different compositions for 7 days. After the intervention, the cells were treated with 0.3% Triton X-100 for 10 min, blocked with 5% BSA for 3 min, and incubated with anti-CD206 (Abcam, USA) and inducible nitric oxide synthase (iNOS, Abcam, USA) primary antibodies at 4°C overnight, followed by incubation with secondary antibodies for 2 h, 100 μL DAPI staining for 5 minutes, air-drying, anti-fluorescence quencher fixation, and image acquisition using an inverted fluorescence microscope. After treating the MWCNT porous scaffolds with different components for 7 days, collect RAW264. 7 cells from each group, extract total RNA from each group of cells using Trizol lysis buffer, and synthesize cDNA. Perform qPCR analysis using the SYBR Green dye method. Finally, GAPDH was used as the housekeeping gene to standardize the qPCR reactions, and the 2^-ΔΔCt^ method was employed to calculate the expression levels of the corresponding genes. Primer sequences are provided in Table S2 of the supplementary materials.

### SONFH rabbit model construction and scaffold implantation

A control group of seven rabbits and a modeling group of seven rabbits were created from the fourteen New Zealand white rabbits. The particular modeling approach was predicated on earlier studies.^67^ SONFH was induced in rabbits using the established lipopolysaccharide (LPS) + methylprednisolone (MPS) protocol. Briefly, rabbits received an intravenous injection of LPS (10 μg/kg) via the marginal ear vein. 24 h later, methylprednisolone (20 mg/kg) was administered intramuscularly once daily for three consecutive days (a total of three injections at 24-h intervals). This protocol has been widely used to induce steroid-associated osteonecrosis in rabbits with a high incidence. Four weeks after induction, osteonecrosis development was verified by histological examination (H&E staining), characterized by empty osteocyte lacunae and necrotic bone marrow changes. Only rabbits with confirmed osteonecrotic lesions were subsequently included for scaffold implantation.^68^ To determine the success rate of bone necrosis modeling, tissue samples were taken for HE staining four weeks after the animals were put to sleep. Following successful modeling, New Zealand white rabbits were split into five groups at random: 12 rabbits in the 0.2% MWCNT group, 12 rabbits in the 0.5% MWCNT group, 12 rabbits in the 1% MWCNT group, 12 rabbits in the nHA group, and 12 rabbits in the blank control group. At seven days, four weeks, and twelve weeks after implantation, the animals were put to sleep, and tissues were taken for histological and imaging examination.

The following is the surgical process for scaffold implantation and medullary decompression: Weigh and anesthetize the New Zealand white rabbits with 3% sodium pentobarbital (injected intraauricularly 30 mg/kg before surgery) and Suoxin II (injected intramuscularly 1 mg/kg during surgery); shave, clean, and cover the bilateral hip-to-knee joint areas; Beginning at the bottom edge of the femur’s greater trochanter’s highest point, make an incision that extends 2–3 cm down the skin and subcutaneous tissue. To reveal the femoral shaft, bluntly remove the pertinent muscles. The K-wire should be inserted using an orthopedic drill (drill bit diameter 3.5 mm) at a 40°–45° angle to the femoral shaft, with an insertion depth of 32–34 mm. The K-wire should be inserted 0.5–1 cm below the lower edge of the greater trochanter. The appropriate scaffold materials should then be immediately implanted into the channel, and the external opening of the channel should be sealed with bone wax. In order to prevent infection, suture the incision, clean the area, and give antibiotics.

### Hard tissue section preparation and analysis

At week 4, New Zealand White rabbits (*n* = 15, 3 per group) were euthanized on the tenth and third days before. Calcium (10 mg/kg, Sigma Aldrich) and xylenol orange (90 mg/kg, Sigma Aldrich) were injected subcutaneously into the gluteal skin. Tissue samples were taken after euthanasia, sectioned, and preserved for 48 h. Method for preparing hard tissue sections: To achieve alcohol gradient dehydration, place the fixed tissue in an E510 dehydrator (50%, 60%, 70%, 80%, 90%, 100%). Each gradient takes one to two days for samples with thicknesses of 1–2 mm and three to four days for samples with thicknesses of 2–3 mm. The number of days required for dehydration increases with sample thickness. After the tissue has been dehydrated, it is immersed in T7200 resin; this process should be done in the dark. For a full day without decalcification, the specimen is kept in a 1:1 solution of methyl methacrylate (MMA) and embedding agent. Embedding and polymerization: To completely cure the embedding medium that has penetrated the tissue, use high-intensity blue light for about 10 h; use a low-intensity light source for about 4 h while keeping the temperature below 40 °C; apply benzoyl peroxide and let it cure for around 12 h.

Slice the specimen into 100 μm thick sections using the EXAKT hard tissue grinding equipment, then take pictures and conduct analysis using a fully automated fluorescence microscope. Determine the rate of bone mineralization deposition using the photos that were taken. The distance between the calcium/xylenol orange fluorescent markers divided by the time between the two injections yields the mineralization deposition rate (MAR, μm/d), which is the rate of new bone deposition.

### Macrophage polarization analysis in vivo

To evaluate the function of implanted scaffolds in the polarization of macrophages in different groups, immunofluorescence staining was performed. Seven days and four weeks after implantation, the animals were euthanized, tissues were collected, and macrophage immunofluorescence staining was performed. To identify M1/M2 subtypes, CCR7 (Abcam) and CD206 (Bioss Bio) antibodies were selected. Femoral head specimens were collected from each group at each time point, decalcified with saturated ethylenediaminetetraacetic acid, dehydrated with graded ethanol, embedded in paraffin, and subsequently subjected to tissue immunofluorescence staining using the same method as above.

### Micro CT analysis

At 4 and 12 weeks post-operation, rabbits were euthanized, and the femoral heads were harvested and fixed in 10% neutral buffered formalin (NBF) for 5 days at room temperature. The samples were then subjected to micro-CT scanning, and 2D cross-sectional images and 3D reconstructions were generated using Analyze 12.0 (AnalyzeDirect, Overland Park, KS, USA). Micro-CT evaluation was performed using a PerkinElmer Quantum GX micro-CT imaging system (PerkinElmer/RevVity, USA). Scanning was conducted in High-Resolution mode (4-min protocol) at 90 kV and 88 μA with a Cu 0.06 mm + Al 0.5 mm filter. The field of view (FOV) was set to 72 mm, yielding a reconstructed isotropic voxel size of 9 μm. The volume of interest (VOI) was defined as a cylindrical defect region (diameter 3.5 mm, height 10 mm) covering the entire defect depth and margins. Because the implanted scaffold (nHA or MWCNT) is radiopaque, newly formed bone was segmented from the scaffold using a dual-threshold segmentation + mask subtraction strategy: T bone = 350–8 000 HU (mineralized tissue = new bone + scaffold) and T scaffold = 3 000–8 000 HU (high-density scaffold), followed by subtraction of the scaffold mask from the mineralized mask to obtain the new-bone-only mask. Here, ‘new bone’ refers to mineralized tissue formed within the predefined cylindrical defect VOI after implantation, quantified by micro-CT segmentation excluding the radiopaque scaffold phase (thresholding + scaffold masking/subtraction). Thresholds were determined based on HU histogram separation and visually verified on representative slices; identical thresholds were applied to all specimens across groups. BV/TV, Tb.Th, Tb.N, and Tb.Sp were calculated based on the new-bone-only mask using 3D algorithms in accordance with Bouxsein et al. guidelines.^55,69^

### Histological analysis

Specimens were gathered when the rabbits were put to death four and twelve weeks after surgery. After being fixed for 48 h in 10% neutral formaldehyde, the specimens were decalcified for four weeks using 10% EDTA. Before being put in embedding cassettes for ethanol gradient dehydration, the fully softened specimens were carefully trimmed, any excess tissue was scraped off, and they were rinsed clean. The dehydrated femurs were then successively placed in xylene solution for 3 h and 70 °C paraffin for 8 h. The samples were put in molds, filled with paraffin, and allowed to cool for 1 h on a cooling platform set at –20 °C. Following rehydration, hematoxylin-eosin (HE) staining and Goldner staining were carried out, followed by standard dehydration, clearing, and mounting. The solidified tissue wax blocks were either sectioned and stained with a section thickness of 5 µm or stored at room temperature.

To evaluate the creation of new bone, the specimens were examined under an optical microscope. Every group of specimens was examined for new bone growth using Image-Pro Plus 6.0 (IPP) image analysis software. Each group’s femoral head wax slices underwent pancreatic enzyme antigen retrieval, gradient ethanol hydration, and xylene dewaxing. After 30 minutes of blocking with goat serum blocking agent, they were added to ALP (1:300), RUNX2 (1:250), OCN (1:200), VEGF (1:200), and CD31 (1:300) antibodies, which were then left overnight at 4 °C. After being reheated the following day, enzyme conjugates and biotinylated secondary antibodies were added one at a time and incubated for 25 minutes at 37 °C. After 20 to 30 seconds of color development with DAB solution, the samples were counterstained with hematoxylin solution for 1 minute and rinsed with running water for 7 minutes. Graded ethanol was used to dehydrate the sections, followed by xylene clearing and neutral resin mounting. Under light microscopy, positive immunohistochemical staining regions or cells look brownish. Image Pro Plus 6.0 software was used for semi-quantitative analysis.

### Transcriptome sequencing analysis

Prior to sequencing, MWCNT or nHA scaffolds were co-cultured with RAW264.7 for four days, lysed using TRIzol reagent, and stored in cryogenic tubes at –80°C. The Illumina PE150 platform (Illumina, USA) was used for RNA sequencing. The million reads per thousand base pairs approach was used to standardize the data. Western blot analysis and RT-qPCR were used to further validate the transcriptome analysis results. Western blot analysis and RT-qPCR followed the same protocols as previously mentioned. AKT and PI3K gene expression was found using RT-qPCR. The ChemiDoc Touch Imaging System (Bio-Rad, California, USA) was used to get Western blot pictures.

### Statistical analysis

Statistical analysis was performed using Graph Prism 7.0 software. The independent samples *t*-test was used to evaluate two sample groups; one-way ANOVA was used to study three or more groups; and the Tukey post-hoc comparison was used to compare groups pairwise. Statistical significance was indicated as *P < 0.05, **P < 0.01 and ***P < 0.001.

### Supplementary Information

This file contains detailed experimental materials and methods, relative elemental content (Table S1), primer sequences (Table S2), and twelve supplementary figures (Fig. S1-S12). The figures provide extended data on MWCNT scaffold characterization, macrophage polarization, in vitro/in vivo assays, biocompatibility, transcriptome analysis, and cellular uptake. Supplementary information accompanies the manuscript on the Bone Research website http://www.nature.com/boneres.

## Supplementary information

Supplementary material

## References

[1] Weinstein, R. S. Glucocorticoid-induced osteonecrosis. *Endocrine* **41**, 183–190 (2012). 22169965 10.1007/s12020-011-9580-0 PMC3712793

[2] Duan, P. et al. Correction: Exosomal miR-1a-3p derived from glucocorticoid-stimulated M1 macrophages promotes the adipogenic differentiation of BMSCs in glucocorticoid-associated osteonecrosis of the femoral head by targeting Cebpz. *J. Nanobiotechnol.* **22**, 694 (2024). 10.1186/s12951-024-02958-8 PMC11552397 39523298

[3] Ma, M. et al. Osteoimmunology and osteonecrosis of the femoral head. *Bone Jt. Res*. **11**, 26–28 (2022). 10.1302/2046-3758.111.BJR-2021-0467.R1 PMC8801166 35045723

[4] Zhang, Q., Sun, W., Li, T. & Liu, F. Polarization behavior of bone macrophage as well as associated osteoimmunity in glucocorticoid-induced osteonecrosis of the femoral head. *J. Inflamm. Res.* **16**, 879–894 (2023). 36891172 10.2147/JIR.S401968 PMC9986469

[5] Kong, N. et al. An injectable self-adaptive polymer as a drug carrier for the treatment of nontraumatic early-stage osteonecrosis of the femoral head. *Bone Res*. **10**, 28 (2022). 35279673 10.1038/s41413-022-00196-y PMC8918325

[6] Tsubosaka, M. et al. Preclinical models for studying corticosteroid-induced osteonecrosis of the femoral head. *J. Biomed. Mater. Res. B Appl. Biomater.* **112**, e35360 (2024). 38247252 10.1002/jbm.b.35360

[7] Quan, H. et al. Application of biomaterials in treating early osteonecrosis of the femoral head: Research progress and future perspectives. *Acta Biomater.* **164**, 15–73 (2023). 37080444 10.1016/j.actbio.2023.04.005

[8] Lai, Y. et al. Porous composite scaffold incorporating osteogenic phytomolecule icariin for promoting skeletal regeneration in challenging osteonecrotic bone in rabbits. *Biomaterials* **153**, 1–13 (2018). 29096397 10.1016/j.biomaterials.2017.10.025

[9] Sun, Y. et al. Polydopamine grafting polyether ether ketone to stabilize growth factor for efficient osteonecrosis repair. *Sci. Rep.* **15**, 3697 (2025). 39880837 10.1038/s41598-025-86965-1 PMC11779900

[10] Long, J. et al. Regulation of osteoimmune microenvironment and osteogenesis by 3D-printed PLAG/black phosphorus scaffolds for bone regeneration. *Adv. Sci.* **10**, e2302539 (2023). 10.1002/advs.202302539 PMC10558667 37616380

[11] Mondal, S. et al. Hydroxyapatite: a journey from biomaterials to advanced functional materials. *Adv. Colloid Interface Sci.* **321**, 103013 (2023). 37839281 10.1016/j.cis.2023.103013

[12] Biedrzycka, A. & Skwarek, E. Composites of hydroxyapatite and their application in adsorption, medicine and as catalysts. *Adv. Colloid Interface Sci.* **334**, 103308 (2024). 39396420 10.1016/j.cis.2024.103308

[13] Zhang, J., Cao, J., Liu, Y. & Zhao, H. Advances in the pathogenesis of steroid-associated osteonecrosis of the femoral head. *Biomolecules* **14**, 667 (2024). 10.3390/biom14060667 PMC11202272 38927070

[14] Li, H. et al. Nitrogen-doped multiwalled carbon nanotubes enhance bone remodeling through immunomodulatory functions. *ACS Appl. Mater. Interfaces* **13**, 25290–25305 (2021). 33908252 10.1021/acsami.1c05437

[15] Ding, K. et al. Multiwalled carbon nanotubes-reprogrammed macrophages facilitate breast cancer metastasis via NBR2/TBX1 Axis. *ACS Nano* **18**, 11103–11119 (2024). 38623806 10.1021/acsnano.3c11651

[16] Lin, R. et al. Multi-walled carbon nanotubes reversing the bone formation of bone marrow stromal cells by activating M2 macrophage polarization. *Regen. Biomater.* **10**, rbad042 (2023). 37274617 10.1093/rb/rbad042 PMC10234760

[17] E Silva, E. P. et al. In vivo study of conductive 3D printed PCL/MWCNTs scaffolds with electrical stimulation for bone tissue engineering. *Bio-Design Manufact.* **4**, 190–202 (2021).

[18] Luo, S. et al. Advances in electroactive biomaterials: Through the lens of electrical stimulation promoting bone regeneration strategy. *J. Orthop. Transl.* **47**, 191–206 (2024). 10.1016/j.jot.2024.06.009 PMC11261049 39040489

[19] Liu, J. et al. Functional cobalt-doped hydrogel scaffold enhances concurrent vascularization and neurogenesis. *J. Nanobiotechnol.* **23**, 179 (2025). 10.1186/s12951-025-03218-z PMC11984231 40205442

[20] Liu, L. et al. Biomimetic bone tissue engineering hydrogel scaffolds constructed using ordered CNTs and HA induce the proliferation and differentiation of BMSCs. *J. Mater. Chem. B* **8**, 558–567 (2020). 31854433 10.1039/c9tb01804b

[21] Shimizu, M. et al. Carbon nanotubes induce bone calcification by bidirectional interaction with osteoblasts. *Adv. Mater.* **24**, 2176–2185 (2012). 22447724 10.1002/adma.201103832

[22] Gupta, B., Sharma, P. K. & Malviya, R. Carbon nanotubes for targeted therapy: safety, efficacy, feasibility and regulatory aspects. *Curr. Pharm. Des.* **30**, 81–99 (2024). 38185892 10.2174/0113816128282085231226065407

[23] Green, D. W., Ben-Nissan, B., Yoon, K. S., Milthorpe, B. & Jung, H. S. Natural and synthetic coral biomineralization for human bone revitalization. *Trends Biotechnol.* **35**, 43–54 (2017). 27889241 10.1016/j.tibtech.2016.10.003

[24] Wan, M. C. et al. Biomaterials from the sea: future building blocks for biomedical applications. *Bioact. Mater.* **6**, 4255–4285 (2021). 33997505 10.1016/j.bioactmat.2021.04.028 PMC8102716

[25] Luo, Z. et al. Coral-inspired bioactive porous adhesive for fractured bone repair. *Adv. Funct. Mater.* **35**, 2507592 (2025).

[26] Ying, G. et al. Bioprinted injectable hierarchically porous gelatin methacryloyl hydrogel constructs with shape-memory properties. *Adv. Funct. Mater.* **30** (2020). 10.1002/adfm.202003740 PMC7941201 33708030

[27] Liu, W. et al. Low-temperature deposition manufacturing: a novel and promising rapid prototyping technology for the fabrication of tissue-engineered scaffold. *Mater. Sci. Eng. C. Mater. Biol. Appl* **70**, 976–982 (2017). 27772729 10.1016/j.msec.2016.04.014

[28] Nie, P. et al. Preparation and tribological properties of polyimide/carboxyl-functionalized multi-walled carbon nanotube nanocomposite films under seawater lubrication. *Tribol. Lett.* **58**, 7 (2015).

[29] Luo, Y., Li, D., Zhao, J., Yang, Z. & Kang, P. In vivo evaluation of porous lithium-doped hydroxyapatite scaffolds for the treatment of bone defect. *Biomed. Mater. Eng.* **29**, 699–721 (2018). 30282329 10.3233/BME-181018

[30] Liang, H. F. et al. Biomimetic structural protein based magnetic responsive scaffold for enhancing bone regeneration by physical stimulation on intracellular calcium homeostasis. *Adv. Health Mater.* **12**, e2301724 (2023). 10.1002/adhm.202301724 37767893

[31] Li, X. R. et al. 3D cryo-printed hierarchical porous scaffolds provide immobilization of surface-functionalized sleep-inspired small extracellular vesicles: synergistic therapeutic strategies for vascularized bone regeneration based on macrophage phenotype modulation and angiogenesis-osteogenesis coupling. *J. Nanobiotechnol.* **22**, 764 (2024). 10.1186/s12951-024-02977-5 PMC11658104 39695679

[32] Tabia, Z., Bricha, M., El Mabrouk, K. & Vaudreuil, S. Manufacturing of a metallic 3D framework coated with a bioglass matrix for implant applications. *J. Mater. Sci.* **56**, 1658–1672 (2020).

[33] Khoramgah, M. S. et al. Freeze-dried multiscale porous nanofibrous three-dimensional scaffolds for bone regeneration. *Bioimpacts* **10**, 73–85 (2020). 32363151 10.34172/bi.2020.10 PMC7186540

[34] Bai, L. et al. Multifunctional scaffold comprising metal-organic framework, hydrogel, and demineralized bone matrix for the treatment of steroid-induced femoral head necrosis. *Small* **21**, e2407758 (2025). 39575484 10.1002/smll.202407758

[35] Ding, T., Kang, W., Li, J., Yu, L. & Ge, S. An in situ tissue engineering scaffold with growth factors combining angiogenesis and osteoimmunomodulatory functions for advanced periodontal bone regeneration. *J. Nanobiotechnol.* **19**, 247 (2021). 10.1186/s12951-021-00992-4 PMC8371786 34404409

[36] Wang, J. et al. BMP-2 functional polypeptides relieve osteolysis via bi-regulating bone formation and resorption coupled with macrophage polarization. *NPJ Regen. Med.* **8**, 6 (2023). 36759627 10.1038/s41536-023-00279-2 PMC9911742

[37] Wu, M. et al. Smart-responsive multifunctional therapeutic system for improved regenerative microenvironment and accelerated bone regeneration via mild photothermal therapy. *Adv. Sci.* **11**, e2304641 (2024). 10.1002/advs.202304641 PMC10787108 37933988

[38] Li, Z. et al. Advances in experimental models of osteonecrosis of the femoral head. *J. Orthop. Transl.* **39**, 88–99 (2023). 10.1016/j.jot.2023.01.003 PMC9931935 36819298

[39] Dempster, D. W. et al. Standardized nomenclature, symbols, and units for bone histomorphometry: a 2012 update of the report of the ASBMR Histomorphometry Nomenclature Committee. *J. Bone Miner. Res.* **28**, 2–17 (2013). 23197339 10.1002/jbmr.1805 PMC3672237

[40] van Gaalen, S. M. et al. Use of fluorochrome labels in in vivo bone tissue engineering research. *Tissue Eng. B Rev.* **16**, 209–217 (2010). 10.1089/ten.TEB.2009.0503 19857045

[41] Meng, X. et al. An impaired healing model of osteochondral defect in papain-induced arthritis. *J. Orthop. Transl.* **26**, 101–110 (2021). 10.1016/j.jot.2020.07.005 PMC7773975 33437629

[42] Mehl, J. et al. External mechanical stability regulates hematoma vascularization in bone healing rather than endothelial YAP/TAZ mechanotransduction. *Adv. Sci. (Weinh.)* **11**, e2307050 (2024). 38273642 10.1002/advs.202307050 PMC10987120

[43] Yu, H. et al. Improved tissue-engineered bone regeneration by endothelial cell-mediated vascularization. *Biomaterials* **30**, 508–517 (2009). 18973938 10.1016/j.biomaterials.2008.09.047

[44] Lin, C. S., Xin, Z. C., Dai, J. & Lue, T. F. Commonly used mesenchymal stem cell markers and tracking labels: Limitations and challenges. *Histol. Histopathol.* **28**, 1109–1116 (2013). 23588700 10.14670/hh-28.1109 PMC3839663

[45] Liang, S. et al. The coupling of reduced type H vessels with unloading-induced bone loss and the protection role of Panax quinquefolium saponin in the male mice. *Bone* **143**, 115712 (2021). 33164873 10.1016/j.bone.2020.115712

[46] Vergadi, E., Ieronymaki, E., Lyroni, K., Vaporidi, K. & Tsatsanis, C. Akt Signaling Pathway in Macrophage Activation and M1/M2 Polarization. *J. Immunol.* **198**, 1006–1014 (2017). 28115590 10.4049/jimmunol.1601515

[47] Covarrubias, A. J., Aksoylar, H. I. & Horng, T. Control of macrophage metabolism and activation by mTOR and Akt signaling. *Semin Immunol.* **27**, 286–296 (2015). 26360589 10.1016/j.smim.2015.08.001 PMC4682888

[48] Luyendyk, J. P. et al. Genetic analysis of the role of the PI3K-Akt pathway in lipopolysaccharide-induced cytokine and tissue factor gene expression in monocytes/macrophages. *J. Immunol.* **180**, 4218–4226 (2008). 18322234 10.4049/jimmunol.180.6.4218 PMC2834303

[49] Ruckerl, D. et al. Induction of IL-4Ralpha-dependent microRNAs identifies PI3K/Akt signaling as essential for IL-4-driven murine macrophage proliferation in vivo. *Blood* **120**, 2307–2316 (2012). 22855601 10.1182/blood-2012-02-408252 PMC3501641

[50] Na, N. et al. Carbamylated erythropoietin regulates immune responses and promotes long-term kidney allograft survival through activation of PI3K/AKT signaling. *Signal Transduct. Target Ther.* **5**, 194 (2020). 32934199 10.1038/s41392-020-00232-5 PMC7493938

[51] Teo, B. K. et al. Nanotopography modulates mechanotransduction of stem cells and induces differentiation through focal adhesion kinase. *ACS Nano* **7**, 4785–4798 (2013). 23672596 10.1021/nn304966z

[52] Liu, Y. C., Zou, X. B., Chai, Y. F. & Yao, Y. M. Macrophage polarization in inflammatory diseases. *Int J. Biol. Sci.* **10**, 520–529 (2014). 24910531 10.7150/ijbs.8879 PMC4046879

[53] Hu, K. & Olsen, B. R. The roles of vascular endothelial growth factor in bone repair and regeneration. *Bone* **91**, 30–38 (2016). 27353702 10.1016/j.bone.2016.06.013 PMC4996701

[54] Parithimarkalaignan, S. & Padmanabhan, T. V. Osseointegration: an update. *J. Indian Prosthodont Soc.* **13**, 2–6 (2013). 24431699 10.1007/s13191-013-0252-z PMC3602536

[55] Bouxsein, M. L. et al. Guidelines for assessment of bone microstructure in rodents using micro-computed tomography. *J. Bone Miner. Res*. **25**, 1468–1486 (2010). 20533309 10.1002/jbmr.141

[56] Qin, L. et al. Phytomolecule icaritin incorporated PLGA/TCP scaffold for steroid-associated osteonecrosis: Proof-of-concept for prevention of hip joint collapse in bipedal emus and mechanistic study in quadrupedal rabbits. *Biomaterials* **59**, 125–143 (2015). 25968462 10.1016/j.biomaterials.2015.04.038 PMC7111223

[57] Chen, Y., Chen, Z., Lei, K., Ding, J. & Yu, L. Surface modification of polyetheretherketone for boosted osseointegration: a review. *Biomater. Transl.* **6**, 181–201 (2025). 40641993 10.12336/bmt.24.00052 PMC12237801

[58] Kinaret, P. A. S., Scala, G., Federico, A., Sund, J. & Greco, D. Carbon nanomaterials promote M1/M2 macrophage activation. *Small* **16**, e1907609 (2020). 32250056 10.1002/smll.201907609

[59] Pei, B., Wang, W., Dunne, N. & Li, X. Applications of carbon nanotubes in bone tissue regeneration and engineering: superiority, concerns, current advancements, and prospects. *Nanomaterials* **9**, 1501 (2019). 10.3390/nano9101501 PMC6835716 31652533

[60] Nazarnezhad, S., Baino, F., Kim, H. W., Webster, T. J. & Kargozar, S. Electrospun nanofibers for improved angiogenesis: promises for tissue engineering applications. *Nanomaterials* **10**, 1609 (2020). 10.3390/nano10081609 PMC7466668 32824491

[61] Rambhia, K. J. & Ma, P. X. Controlled drug release for tissue engineering. *J. Control Release* **219**, 119–128 (2015). 26325405 10.1016/j.jconrel.2015.08.049 PMC4656104

[62] Pierce, T. P. et al. A current review of core decompression in the treatment of osteonecrosis of the femoral head. *Curr. Rev. Musculoskelet. Med*. **8**, 228–232 (2015). 26045085 10.1007/s12178-015-9280-0 PMC4596206

[63] Karageorgiou, V. & Kaplan, D. Porosity of 3D biomaterial scaffolds and osteogenesis. *Biomaterials* **26**, 5474–5491 (2005). 15860204 10.1016/j.biomaterials.2005.02.002

[64] Singh, R. et al. Engineered nanomaterials for immunomodulation: a review. *ACS Appl. Bio. Mater.* **7**, 727–751 (2024). 38166376 10.1021/acsabm.3c00940

[65] Hosseini, F. S. et al. Graphene oxide in bone regenerative engineering: current challenges and future perspectives. *ACS Bio. Med. Chem. Au* **5**, 350–364 (2025). 40556777 10.1021/acsbiomedchemau.4c00152 PMC12183520

[66] Petek, D., Hannouche, D. & Suva, D. Osteonecrosis of the femoral head: pathophysiology and current concepts of treatment. *EFORT Open Rev.* **4**, 85–97 (2019). 30993010 10.1302/2058-5241.4.180036 PMC6440301

[67] Li, D. et al. Delivery of MiR335-5p-pendant Tetrahedron DNA nanostructures using an injectable heparin lithium hydrogel for challenging bone defects in steroid-associated osteonecrosis. *Adv. Health Mater.* **11**, e2101412 (2022). 10.1002/adhm.202101412 34694067

[68] Qin, L. et al. Multiple bioimaging modalities in evaluation of an experimental osteonecrosis induced by a combination of lipopolysaccharide and methylprednisolone. *Bone* **39**, 863–871 (2006). 16765664 10.1016/j.bone.2006.04.018 PMC7103395

[69] Roy, A. et al. De novo design of highly selective miniprotein inhibitors of integrins alphavbeta6 and alphavbeta8. *Nat. Commun.* **14**, 5660 (2023). 37704610 10.1038/s41467-023-41272-z PMC10500007

