# Supplementary material for: Coral-inspired immunoreprogramming scaffold reverses the “immune-freeze” microenvironment to promote bone regeneration in steroid-induced osteonecrosis of the femoral head
Source: Bone Res. 2026 Jun 30;14:69. doi: 10.1038/s41413-026-00557-x (PMC13319735; doi:10.1038/s41413-026-00557-x)
Supplement: Supplementary file 1 — Supplementary material [file 41413_2026_557_MOESM1_ESM.pdf]

Supplementary Information for
Coral-Inspired Immunoreprogramming Scaffold Reverses the "Immune-Freeze" Microenvironment to Promote Bone Regeneration in Steroid-Induced Osteonecrosis of the Femoral Head

Yue Luo, Qianhao Li *et al.*

Supplementary Text

Materials and Methods

MWCNT bionic scaffolds mediate macrophage activation for osteogenic differentiation

500 μL of the cell suspension should be inoculated into the upper chamber of a transwell chamber (α-MEM medium) after 1×10^5 L^{-1} RAW264.7 cells have been pre-treated with interferon-γ + lipopolysaccharide + dexamethasone. A 12-well plate (α-MEM medium) was seeded with a 1×10^6 L^{-1} bone marrow mesenchymal stem cell solution (1.5 mL). Both components were then incubated overnight, and MWCNT scaffolds of various compositions were introduced to the upper chamber. Following their transfer to the 12-well plate containing bone marrow mesenchymal stem cells, the chambers containing RAW264.7 cells and various scaffolds were co-cultured in an incubator for seven days. After seven days of co-cultivation, the upper chamber was taken out, the 12-well plate's lower chamber was cleaned with PBS, and tests for osteogenic induction, cell scratch migration, and immunofluorescence labeling were carried out. Western blot and RT-qPCR techniques were used to identify osteogenic marker genes and proteins in bone marrow mesenchymal stem cells. Table S2 in the supplemental materials contains a list of the primer sequences.

Alkaline phosphatase staining: Following seven days of co-culturing, BMSCs were stimulated to differentiate into osteoblasts using osteogenic induction differentiation media, which was changed every two days for a total of seven days. Using an inverted phase-contrast microscope, observe and take pictures after fixing the cells with 40 g/L paraformaldehyde for 30 minutes at room temperature, washing them twice with PBS, making the BCIP/NBT staining working solution as directed, staining them for 5 minutes at room temperature, and then rinsing them three times with PBS.

Alizarin red staining: Use osteogenic induction differentiation medium to co-culture BMSCs for 7 days, switching it out every 3 days for a total of 14 days. Using an inverted phase-contrast microscope, examine and take pictures after fixing the cells with 40 g/L paraformaldehyde for 30 minutes at room temperature, washing them twice with PBS, staining them with 0.1% alizarin red S staining solution for 30 minutes at room temperature, and then washing them three times with PBS.

The cell scratch assay involves seeding BMSCs cell suspension into a well plate. After the cells have grown confluently, draw a straight line on the dish's bottom using a ruler and a 1000 μl pipette tip. Continue cultivating in a cell incubator after washing away cell debris with PBS and adding various interventions. Examine cellular alterations under a microscope and capture images 24 hours later; determine the rate of cell migration with ImageJ software. The average scratch width is equal to the scratch gap area divided by the length, and the cell migration rate is equal to (0 h scratch width - scratch width after 24 h of culture) / 0 h scratch width × 100%.

Cell immunofluorescence: A 12-well plate with embedded coverslips was seeded with 1 × 10^6 L-1BMSCs cell suspension (1 mL) and incubated for 24 hours. Then co-culture in an incubator for seven days after adding RAW264.7 and various scaffold treatments to the upper chamber. Remove the cell slides and upper chamber after the seven-day co-cultivation period. 4% paraformaldehyde is used to fix the cell slides, 0.5% Triton X-100 permeation and 1% bovine serum albumin blocking are used to block them, and then BMP2 primary antibody working solution is added and the cells are incubated at 4°C for the entire night. The following day, the cell monolayer is cleaned, and then the secondary antibody working solution is added in the dark and incubated for two hours (at 37°C). After 30 minutes of room temperature dark incubation, add the Actin-Tracker Green staining working solution to the slide. Then, after 10 minutes of dark washing with 0.1% Triton X-100, add the DAPI working solution and incubate for another 10 minutes. Finally, cover the slide with PBS, examine the cells under a laser confocal microscope, and take pictures.

RT-q PCR technique: Using the particular technique previously mentioned, the expression levels of osteogenic marker genes (ALP, COL-1, Runx2, and OPN) mRNA in BMSCs were determined using the fluorescence real-time quantitative polymerase chain reaction (q RT-PCR). Western blot analysis: Each set of BMSC cell culture plates should include 100 μL of protein lysis solution. The cells should then be lysed on ice for 30 minutes, centrifuged at 12,000 rpm for 10 minutes at 4°C, the supernatant collected, and stored at -80°C. Ascertain the protein concentration, make a gel, fill each well with 10 μL, conduct constant-voltage electrophoresis, activate the PVDF membrane with methanol for one minute, and then arrange the filter paper (anode) in the electrophoresis chamber. -PVDF membrane, gel, and filter paper (cathode) conduct electrotransfer for two hours at a steady 200 mA current; the primary antibodies, Runx2 (1:500), COL-1 (1:500), and BMP2 (1:1,000), are blocked with 5% BSA for one hour. The secondary antibodies are then incubated for one hour at 4°C. Use a chemiluminescence device to expose and maintain the protein bands, and use the ImageJ quantitative analysis software to measure the outcomes.

MWCNT bionic scaffolds mediate angiogenesis through macrophage activation

Before inoculating 500 μL of the 1 × 10^5 L^{-1} RAW264.7 cell suspension with interferon-γ, lipopolysaccharide, and dexamethasone, the upper chamber of the transwell chamber (α-MEM medium) should be prepared. A 12-well plate (α-MEM medium) was seeded with a 1×10^6 L-1 HUVECs cell suspension (1.5 mL). Both sections were then incubated overnight, and MWCNT porous scaffolds of various compositions were introduced to the upper chamber. Following their transfer to the 12-well plate containing HUVEC cells, the chambers containing RAW264.7 cells

and various scaffolds were co-cultured in an incubator for seven days. Following seven days of co-culturing, the upper chamber was taken out, the 12-well plate's lower chamber was cleaned with PBS, and immunofluorescence staining, tube formation, and cell scratch migration tests were carried out. Following the collection of HUVEC cells, RT-qPCR was used to identify osteogenic marker genes. Table S2 in the supplemental materials displays the primer sequences.

Assay for HUVEC tube formation: After transferring the cell chambers seeded with various scaffolds and RAW264.7 to a 12-well plate, place them in an incubator set at 37°C. To a 24-well plate, add 100 μL of Matrigel gel to each well. After 30 minutes of incubation at 37°C, add conditioned media that contains roughly 6×10^4 HUVECs per well. Then, continue the culture. As a negative control, use standard DMEM media. After four hours of culture, use an optical microscope to view the in vitro tube creation of endothelial cells. Then, use ImageJ software to determine the total length of the tubes and the number of vascular crossings.

The cell scratch experiment involves seeding a well plate with HUVEC cell suspension, then using a ruler and a 1000 μl pipette tip to draw a straight line on the dish's bottom once the cells have grown confluently. Continue cultivating in a cell incubator after washing away cell debris with PBS and adding various interventions. Cell migration rate was calculated using ImageJ software: cell migration rate = (0 h scratch width - scratch width after 24 h of culture) / 0 h scratch width × 100%, average scratch width = scratch gap area / length. Cell changes were observed under a microscope, and photographs were taken after 24 hours.

Immunofluorescence mediated by cells: 1×10^6 L-1 HUVECs cell suspension A 12-well plate with integrated coverslips was seeded with 1 mL, and it was cultured for 24 hours. Then co-culture in an incubator for seven days after adding RAW264.7 and various scaffold treatments to the upper chamber. Remove the cell coverslip and upper chamber after the seven-day co-cultivation period. After fixing the cell coverslip with 4% paraformaldehyde, blocking it with 0.5% Triton X-100 permeabilization and blocking it with 1% bovine serum albumin, adding the VEGF primary antibody working solution, and incubating it at 4°C for the entire night, the cell monolayer was cleaned the following day, and the secondary antibody working solution was added and left in the dark for two hours (at 37°C); the slide should be stained with Actin-Tracker Green staining solution and left in the dark for 30 minutes at room temperature. It should then be cleaned with 0.1% Triton X-100 and left in the dark for 10 minutes. Finally, it should be sealed with PBS and examined under a laser confocal microscope to observe the cells and take pictures.

RT-qPCR technique: Using the particular technique previously mentioned, the expression levels of HUVEC angiogenesis marker genes (MMP2, MMP9) mRNA were determined using the fluorescence real-time quantitative polymerase chain reaction (q RT-PCR).

Rat subcutaneous air-pouch model (air-pouch) construction and implantation of scaffolds

Twelve SD rats were split equally into four groups: four each of the following groups: four in the 0.2% MWCNT group, four in the 0.5% MWCNT group, four in the 1% MWCNT group, and four in the nHA group. The following is the precise modeling technique: The rats should first be weighed and given an intraperitoneal dose of anesthesia. After shaving the rats' backs, clean the area. In order to create a dorsal air bladder, subcutaneously inject 20 mL of sterile air into the SD

rats' dorsal skin. Maintain the air bladder by injecting 10 mL of sterile air on days 3 and 5, respectively. After the air bladder has stabilized for seven days, anesthetize the rats intraperitoneally, clean the area, make a 1 cm incision at the edge of the air bladder, and implant the scaffold for the appropriate group. Finally, close the incision and disinfect the area. The rats were put to sleep seven days after implantation, and their tissues were taken out for hematoxylin and eosin (HE) staining and macrophage immunofluorescence staining. A midline incision was made on the back; the rats were euthanized by cervical dislocation seven days later; the air sac was removed completely, fixed in 4% formaldehyde for a week, dehydrated, cleared, embedded in paraffin, sectioned into slices that were 5 μm thick, stained with standard HE, and examined under a light microscope.

Immunofluorescence staining: Graded ethanol was used to hydrate paraffin slices of air sacs from each group after they had been dewaxed with xylene. Following sodium citrate antigen repair, they were blocked for 30 minutes with goat serum blocking agent, treated with prepared primary antibody working solution (F4/80, CD206, iNOS), and then incubated for an entire night at 4°C in a refrigerator; fluorescence secondary antibody (1:100) was added, and they were incubated for 1–2 hours at 37°C in the dark; DAPI staining solution was added, and they were incubated for 15 minutes at room temperature in the dark. Mount the slide once it has slightly dried, then use ImageJ software to examine the fluorescence expression under a microscope.

Transmission electron microscopy (TEM) for cellular internalization of MWCNTs

For TEM observation of cellular internalization, RAW264.7 macrophages (and rBMSCs, if applicable) were incubated with MWCNT-containing scaffold extracts for 24 h, fixed in 2.5% glutaraldehyde, post-fixed in 1% osmium tetroxide, dehydrated through graded ethanol, embedded in resin, and sectioned into ultrathin slices. Sections were stained with uranyl acetate and lead citrate before imaging by TEM.

Results

1. Characterization of MWCNT scaffolds

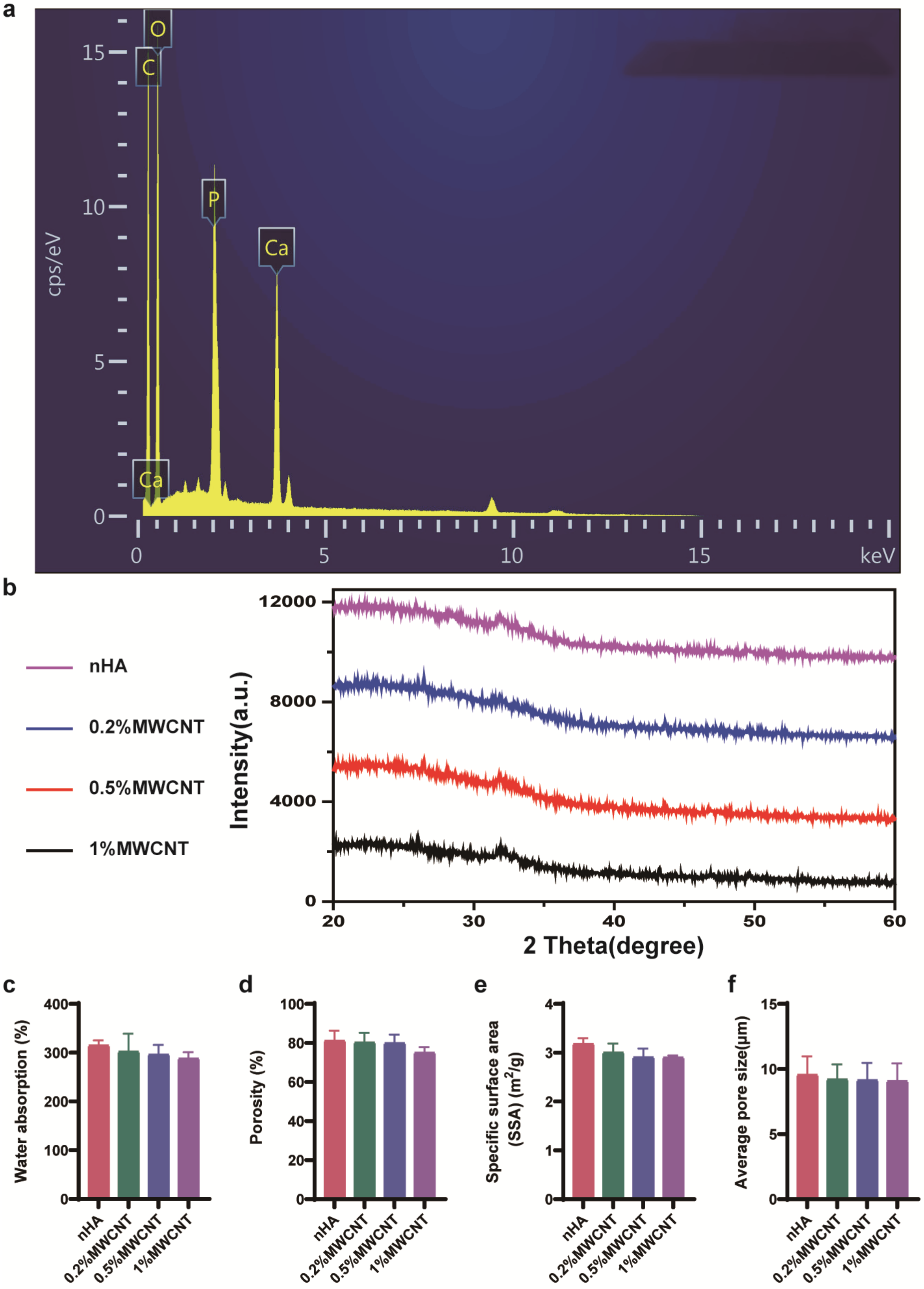

Fig. S1. Physicochemical characterization of MWCNT bionic scaffolds. a: Elemental distribution by SEM-EDS. b: X-Ray Diffraction (XRD) patterns of MWCNT bionic scaffolds with different compositions. c: Results of water absorption of MWCNT bionic scaffolds with different compositions. d: Porosity size of each group of porous scaffolds. e: Specific surface area (SSA) of each group of porous scaffolds. f: Average pore size of each group of scaffolds.

In the pore size distribution measurements, most of the generated pore size distributions were in the range of 2-16 μm, with an average pore size of 9 μm **(Fig. S2)**.

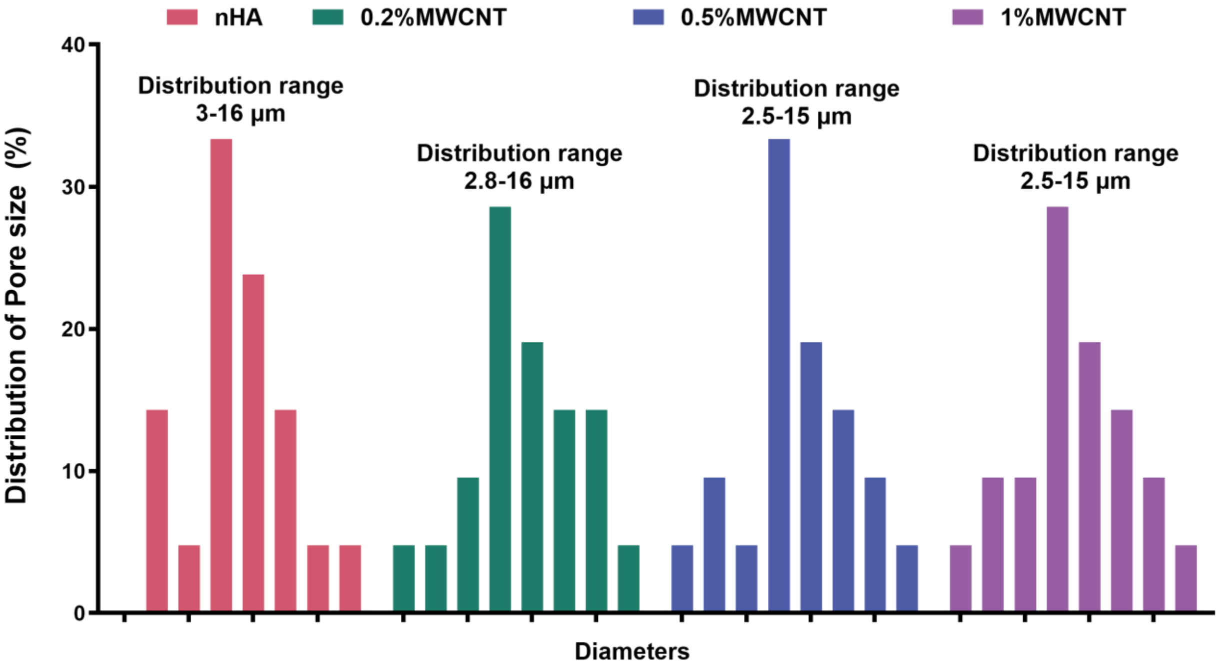

Fig. S2. Distribution of pore sizes in each group of porous scaffolds.

2. Effect of MWCNT on the polarization state of macrophages under the action of GCs

In order to clarify the effects of GCs ($5X10^{-6}$ mol/L), GCs+LPS+IFN-γ, LPS (100 ng/ml) +IFN-γ (20 ng/mL), Control on the phenotypic changes of RAW264.7 cells, this study was evaluated using immunofluorescence staining, flow cytometry and ELISA. After 24 h of culture, immunofluorescence staining was performed to detect the expression of F4/80 (red), CCR7 (green) and CD206 (green) in RAW264.7 cells. As shown in **Fig S3a-3b**, the trend of $F4/80^{+}CD206^{+}$ expression was: control> GCs > LPS+IFN-γ> GCs+LPS+IFN-γ. However, $F4/80^{+}iNOS^{+}$ showed the opposite trend: more $F4/80^{+}iNOS^{+}$ positive cells were detected in GCs+LPS+IFN-γ group.

Flow cytometry data (**Fig S4a-4b**) showed that GCs, LPS+IFN-γ and GCs+LPS+IFN-γ polarized more M1 macrophages ($F4/80^{+}CCR7^{+}$cells) and fewer M2 macrophages ($F4/80^{+}CD163^{+}$ cells) compared to Control. Immune-related cytokines were detected by ELISA to further investigate the inflammatory response. The results showed that pro-inflammatory cytokines (TNF-α and IL-6) and anti-inflammatory cytokines (IL-4 and IL-10) were up-regulated and down-

regulated by GCs, LPS+IFN-γ, and GCs+LPS+IFN-γ, compared with Control (**Fig S4c**). The above studies showed that GCs, LPS+IFN-γ, and GCs+LPS+IFN-γ all promoted M0 to M1 cell polarization, with the strongest promotional effect being GCs+LPS+IFN-γ, followed by LPS+IFN-γ. Therefore, in order to better mimic the inflammatory environment of hormonal osteonecrosis in vitro, the GCs+LPS+IFN-γ drug combination was selected in this study for subsequent cellular studies.

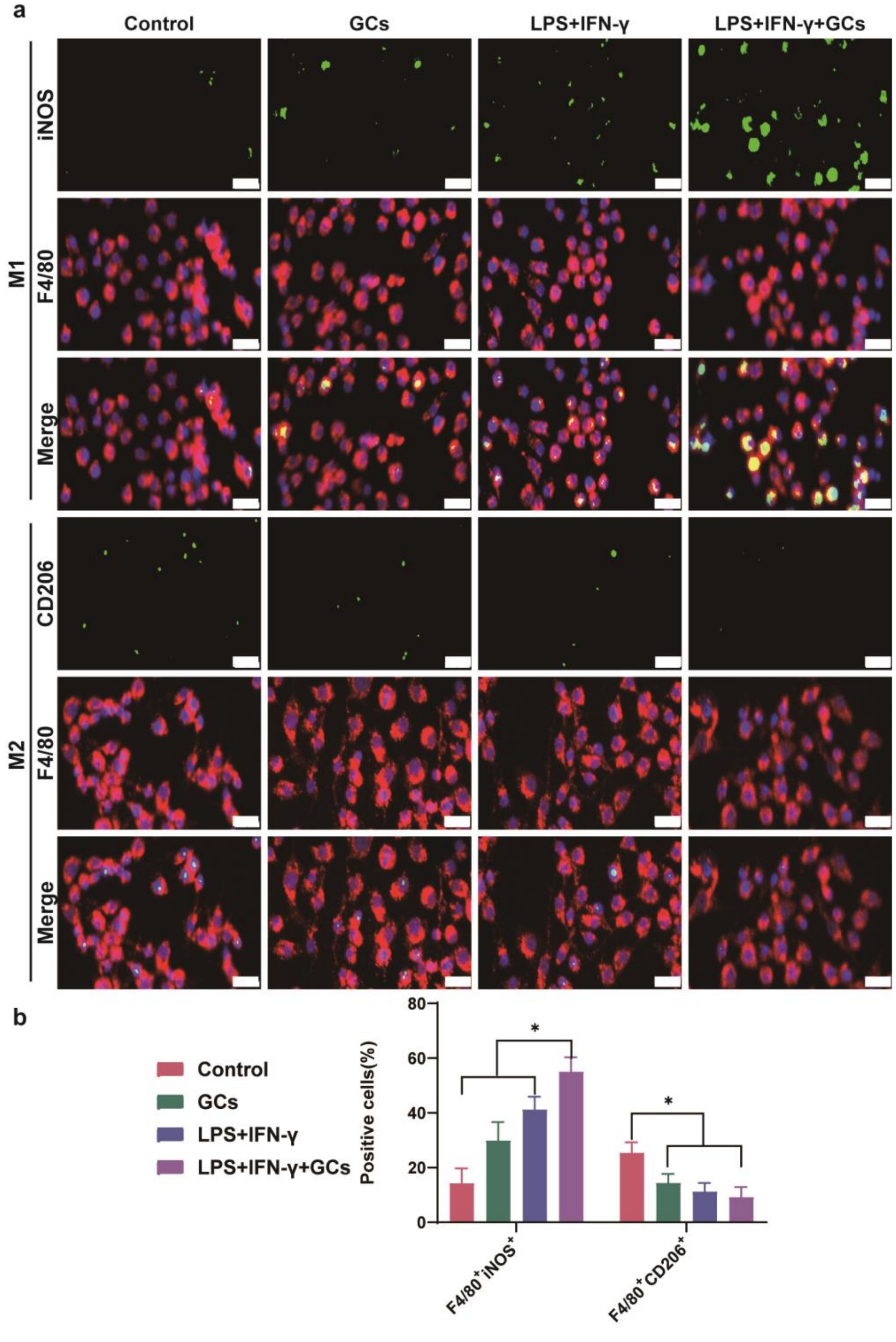

Fig. S3. a-b: RAW264.7 immunofluorescence staining (a) and semi-quantitative analysis results (b). Scale bar is 20 μm. * $p < 0.05$; ** $p < 0.01$; *** $p < 0.001$.

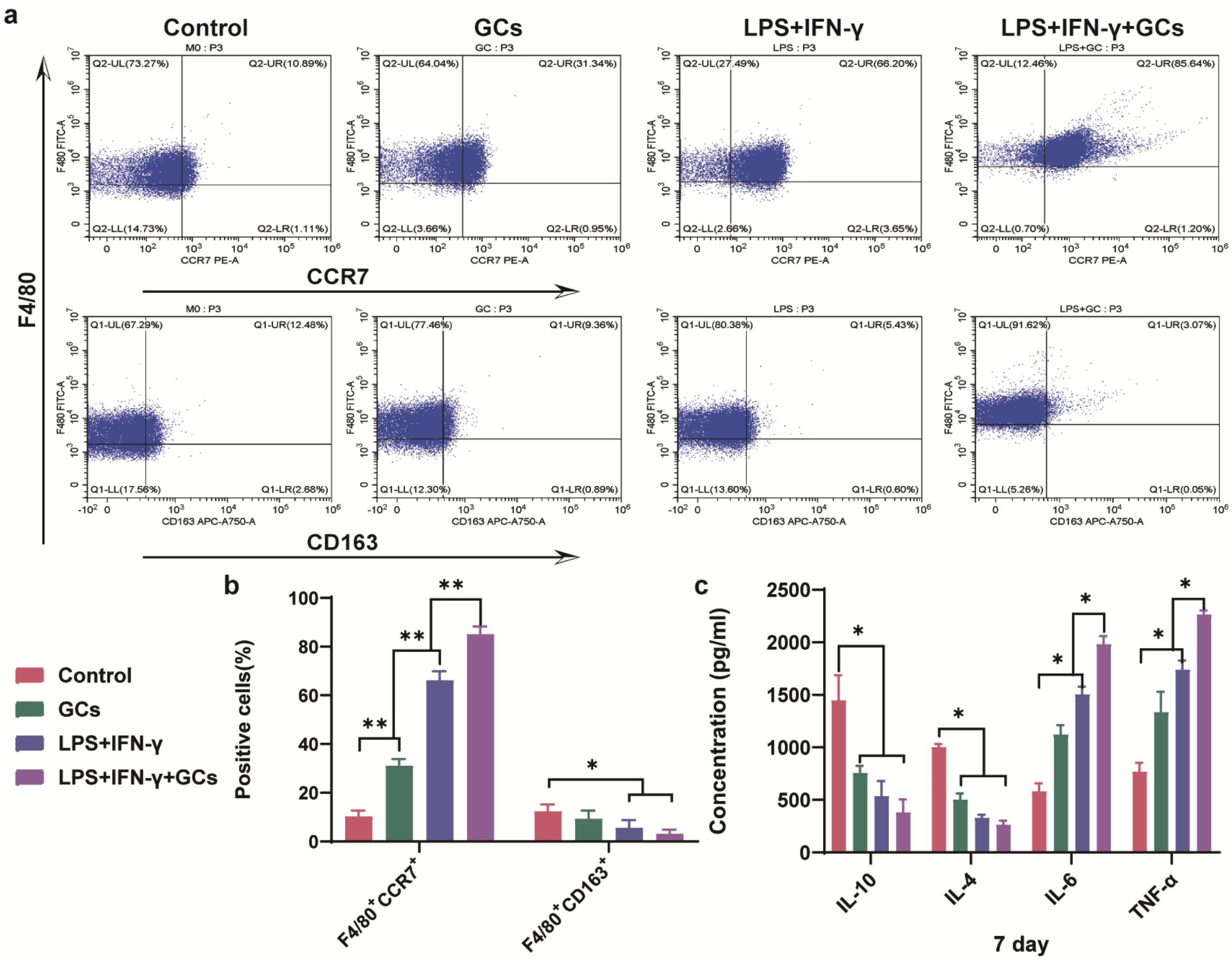

Fig. S4. a-b: RAW264.7 flow cytometric analysis graph (a) and semi-quantitative analysis results (b). c: ELISA to determine the expression of cytokines of RAW264.7 under each group of treatment. * $p < 0.05$; ** $p < 0.01$; *** $p < 0.001$.

3. MWCNT bionic scaffolds mediate macrophage activation for osteogenic differentiation

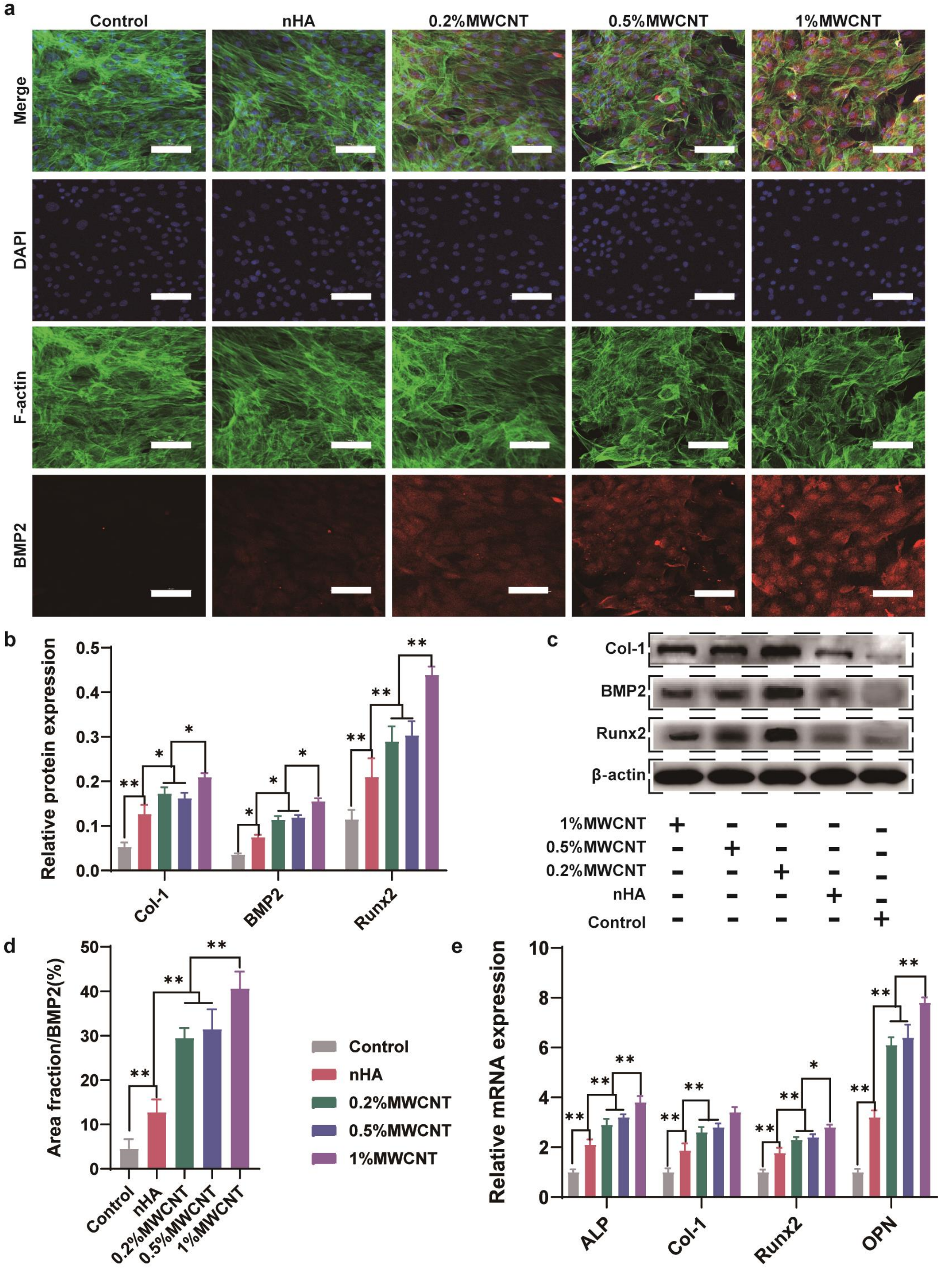

Fig. S5. MWCNT bionic scaffolds of different compositions mediate macrophage regulation of rBMSCs osteogenic differentiation. a and d: Immunofluorescence staining images of BMP2 (BMP2 is red, F-actin is green, and DAPI is blue) and semi-quantitative results (d). The scale bar is 100 μm. b-c: Western-blot detection of the expression of osteogenesis-related proteins in rBMSCs (c) and semi-quantitative analysis of COL-1, BMP2 and Runx2(b). e: Osteogenesis-related gene expression (ALP, Runx2, COL-1, and OPN) in rBMSCs was detected by RT-PCR. * p < 0.05; ** p < 0.01; *** p < 0.001.

4. MWCNT bionic scaffolds mediate angiogenesis through macrophage activation

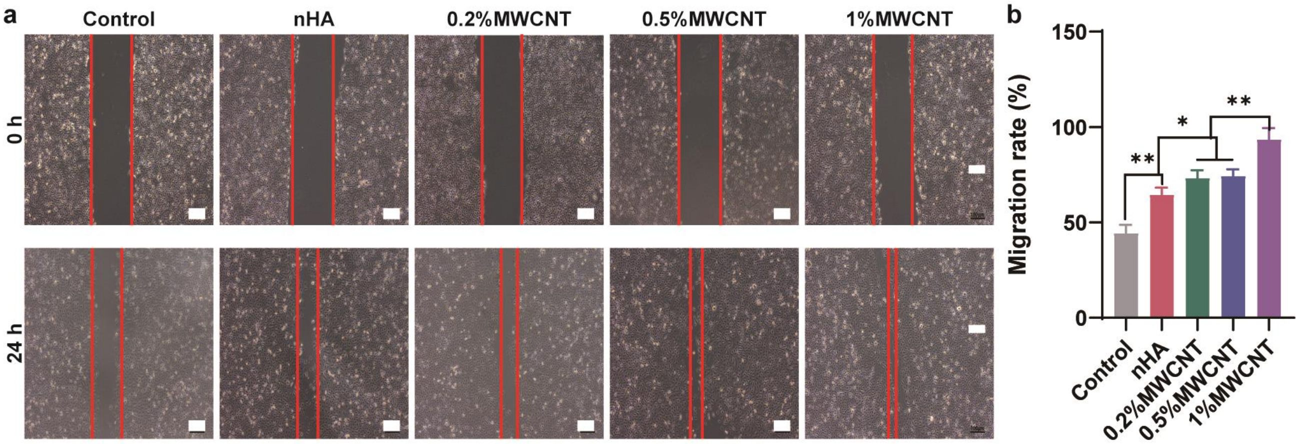

Fig. S6. Macrophage regulation of new vessel development in HUVECs is mediated by MWCNT bionic scaffolds. a: Outcomes of HUVECs scratch tests conducted in the Transwell co-culture system after pretreatment. There is a 100 μm scale bar. b: HUVECs scratch assay cell migration rate analysis. * p < 0.05; ** p < 0.01; *** p < 0.001.

5. In vivo immunoreactivity of MWCNT bionic scaffolds

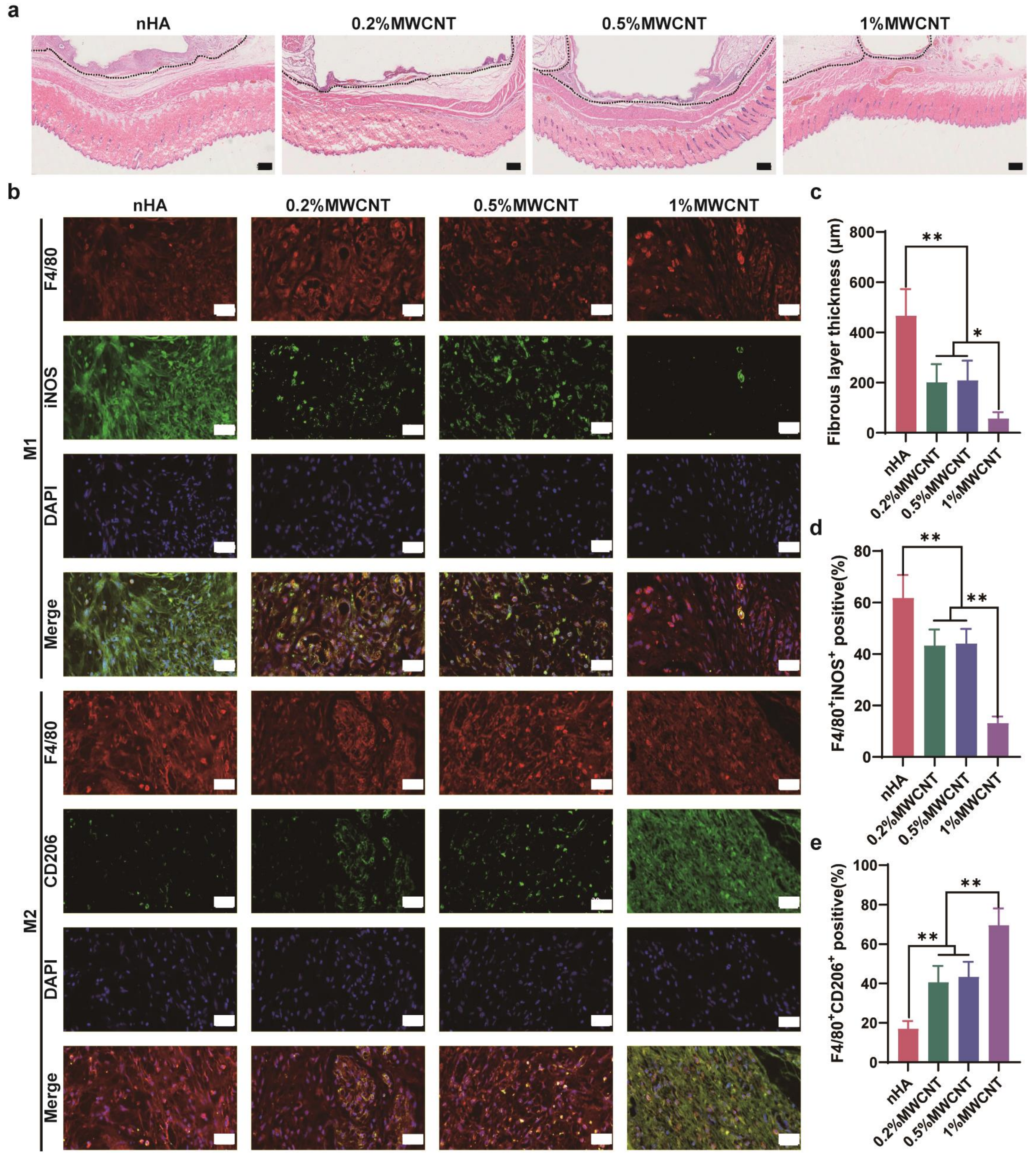

Fig. S7: A rat subcutaneous air pouch paradigm was used to assess in vivo immune responses to MWCNT bionic scaffolds. a and c: Rat subcutaneous air pouch skin (a) stained with HE and quantitative measurement of the fibrous layer thickness (c); the fibrous layer is indicated by the dashed line. There is a 500 μm scale bar. b: $F4/80^+CD206^+$ and $F4/80^+iNOS^+$ immunofluorescence staining in rat subcutaneous air pouch skin (nuclei are blue, F4/80 is red, and CD206 and iNOS are green). The scale bar is 20 μm. d–e: Immunofluorescence staining quantitative analysis. * $p < 0.05$; ** $p < 0.01$; *** $p < 0.001$.

6. Evaluation of a rabbit model of hormonal necrosis of the femoral head (SONFH)

All New Zealand Large White rabbits in the model group survived. HE staining showed that the control group had abundant hematopoietic cells, orderly bone trabeculae, surrounded by a large number of osteoblasts, and a small number of empty bone trabeculae **(Fig. S8a)**. In contrast, the model group showed typical bone necrosis, with a large number of adipocytes with a large diameter in the bone marrow, a large number of empty bone trabeculae in the bone trabeculae, disordered trabecular arrangement, and part of the trabeculae were broken **(Fig. S8a)**. The rate of empty bone traps in the control group was (17.34±6.65)%, and the rate of empty bone traps in the model group was (63.75±8.73)%, which was significantly higher compared with that of the control group **(Fig. S8b)**, and the necrosis rate in the model group was 78.57%.

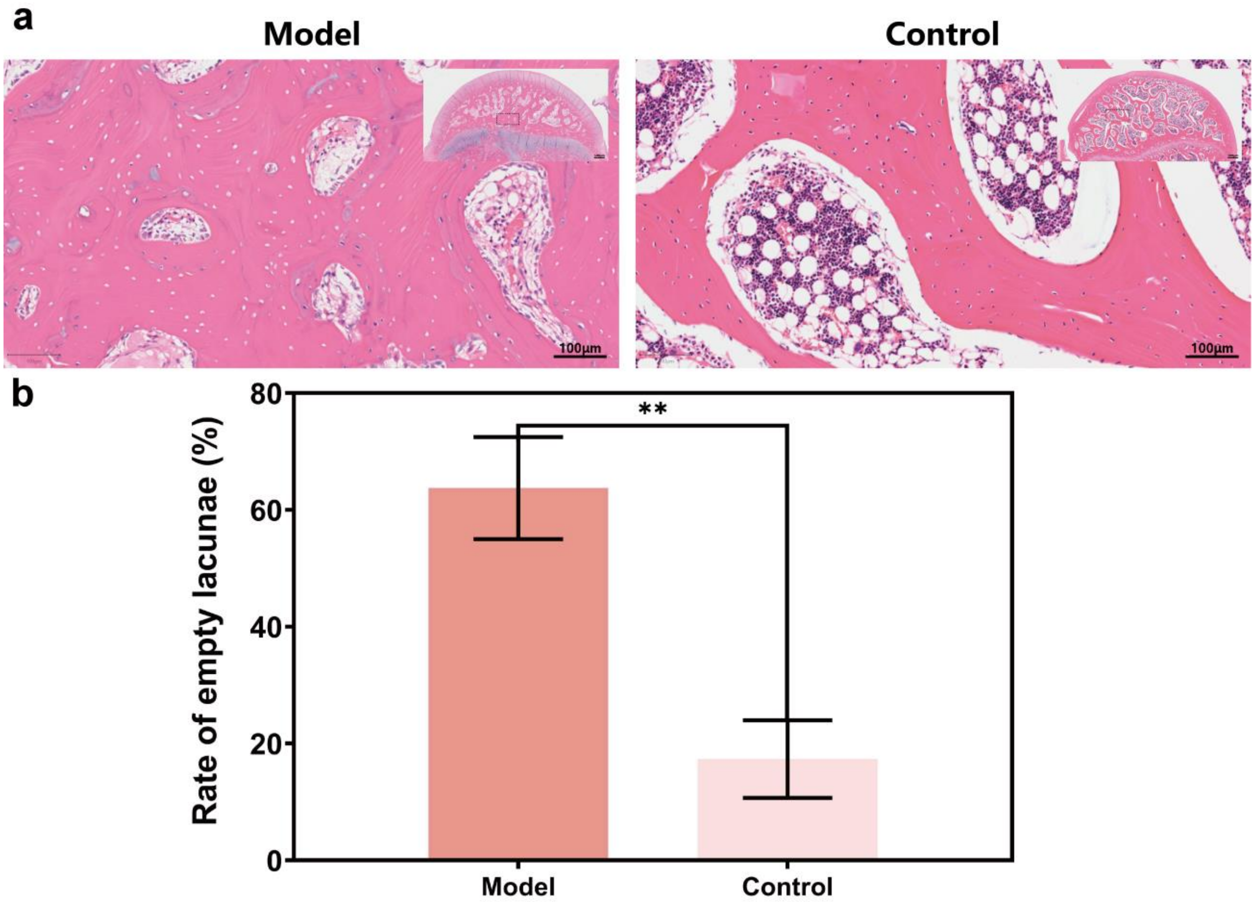

Fig. S8. a: HE staining results of tissue sections from normal and ONFH model groups. The scale bar was 500 μm,100 μm. b: Analysis of the rate of empty lacunae in both normal and ONFH model groups. * $p < 0.05$; ** $p < 0.01$; *** $p < 0.001$.

7. New Bone Formation by Histologic Analysis

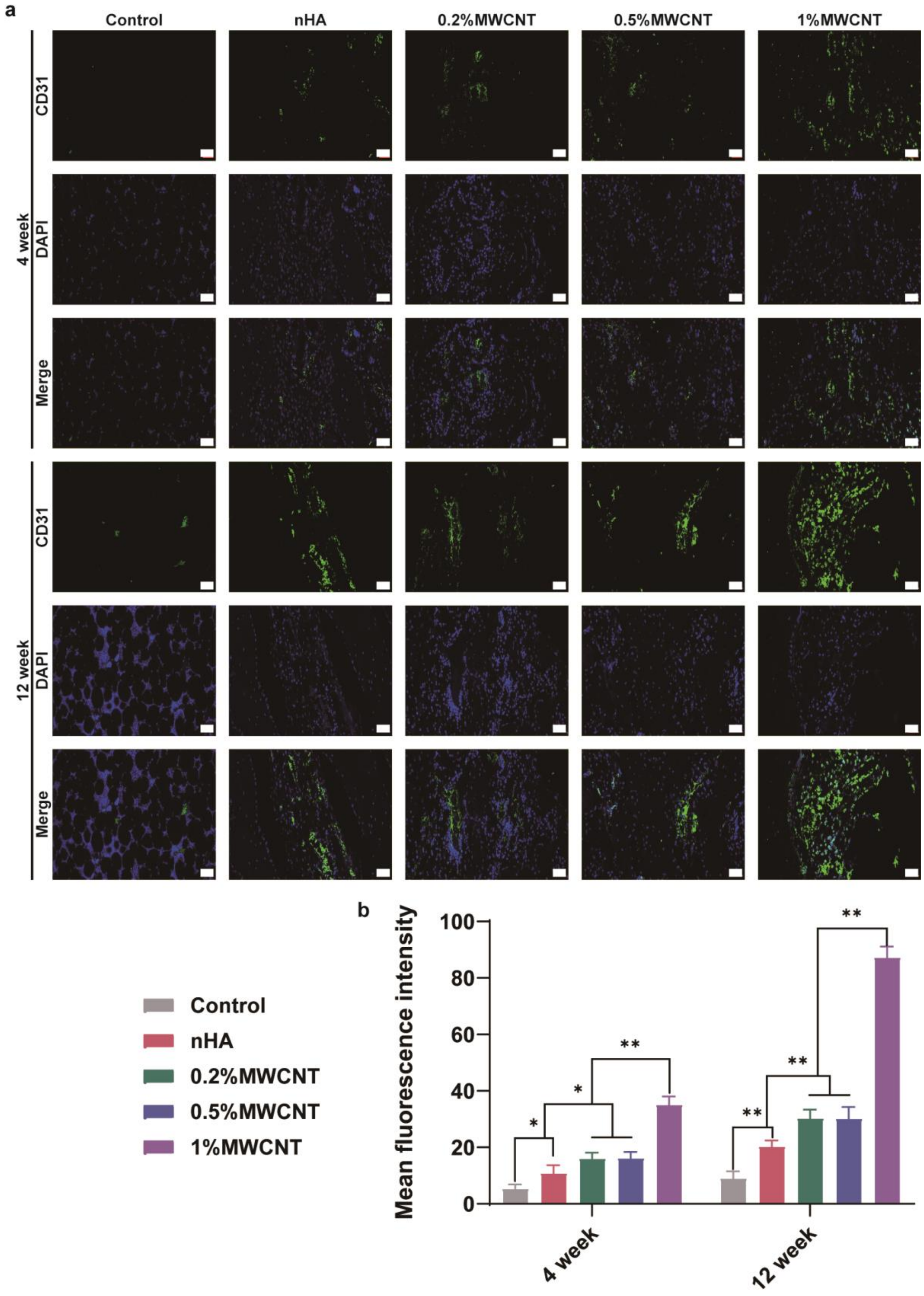

Fig. S9. a: Immunofluorescence staining showing the expression of CD31 (green) in the bone

defect area 4 and 12 weeks after scaffold implantation. The cell nuclei were stained with DAPI (blue). The scale bar is 50 μm. b: Quantitative analysis of fluorescence intensity of CD31-positive stained regions using ImageJ software. * $p < 0.05$; ** $p < 0.01$; *** $p < 0.001$.

8. In vivo toxicity assay evaluation and transcriptome analysis of MWCNT porous scaffolds

Four weeks after scaffold implantation, the heart, liver, kidney and spleen of rabbits were sampled for HE staining to evaluate the biocompatibility of MWCNT porous scaffolds in vivo. The results of HE staining showed that the histological structure and cellular morphology of the heart, liver, kidney and spleen of rabbits were normal, and there was no inflammatory infiltration and other abnormal pathologic changes, which indicated that the scaffolds in each group did not have chronic cytotoxicity **(Fig. S10a)**. To further evaluate long-term biosafety, major organs (heart, liver, spleen and kidney) were harvested at 12 weeks post-implantation and subjected to H&E staining. Histological examination showed preserved tissue architecture with no obvious chronic inflammatory cell infiltration, granuloma-like reactions, or fibrotic-like changes among groups, suggesting favorable long-term systemic biocompatibility of the MWCNT porous scaffolds **(Fig. S10b)**. The results demonstrated that the porous scaffolds had good biocompatibility in vivo.

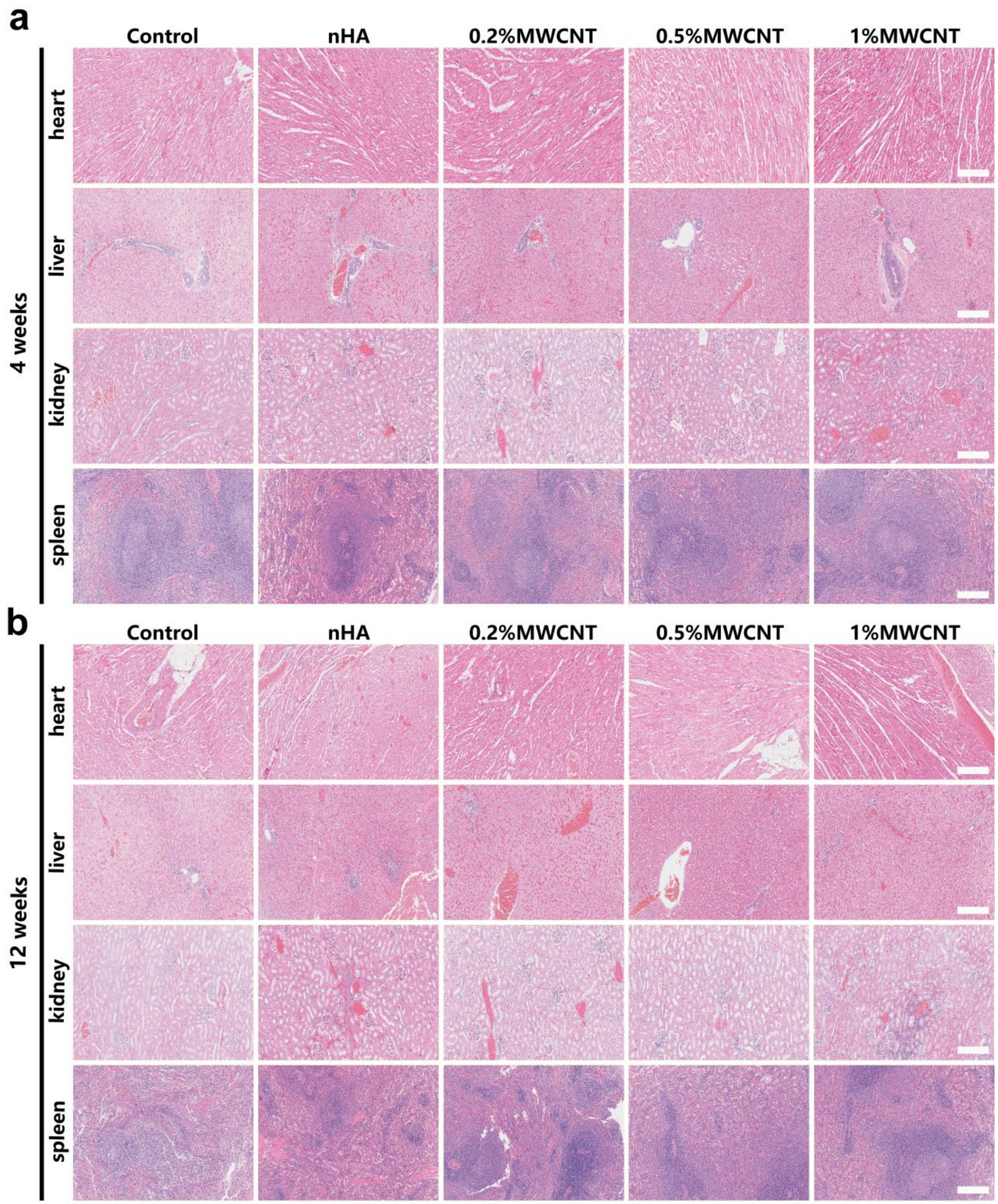

Fig. S10. MWCNT porous scaffold in vivo toxicity evaluation. a: H&E stained histological sections of various tissues of rabbits 4 weeks after implantation of different scaffolds. b: H&E stained histological sections of various tissues of rabbits 12 weeks after implantation of different scaffolds. Scale bar is 50μm.

9. Transcriptome Sequencing analysis results

As shown in the Wayne diagram of **Fig. S11a**, the co-expressed genes in the MWCNT scaffold group and the nHA scaffold group were 19,638 genes, and the number of specifically expressed genes was 2,120 and 1,715 genes, respectively. The GO (Gene Ontology) functional enrichment analysis includes three parts: molecular function, biological process and cellular component (**Fig. S11b**). The 30 terms with the highest number of differentially enriched genes between MWCNT scaffolds and nHA scaffolds included positive regulation of programmed cell death, positive regulation of programmed cell death, positive regulation of immune response, inflammatory response, regulation of cell adhesion, and cellular regulation of cellular response to stress, and actin cytoskeleton organization, which is associated with macrophage polarization.

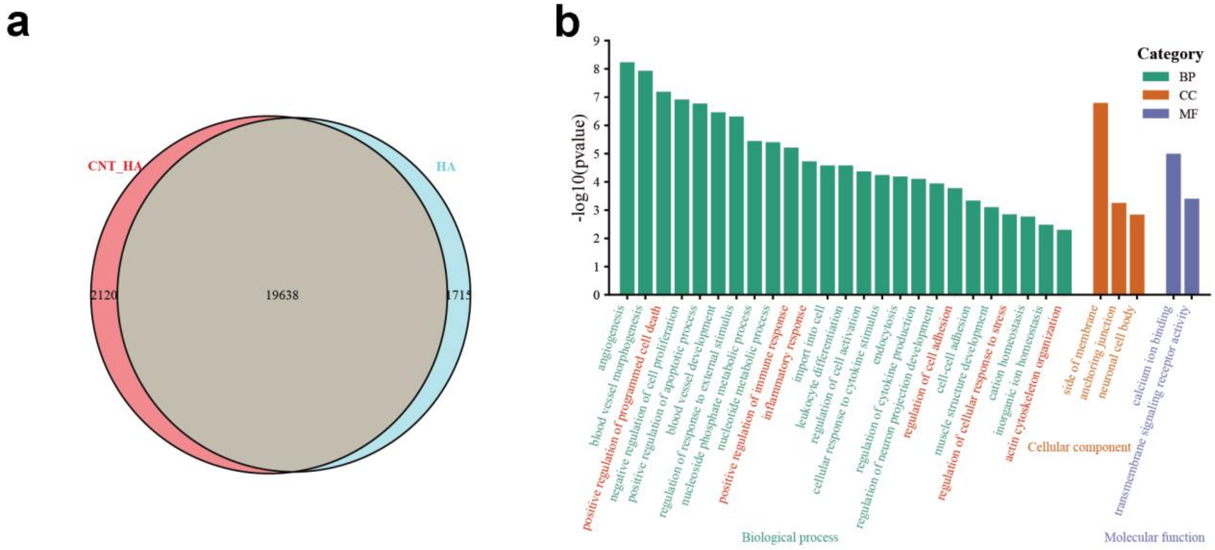

Fig. S11. a: Venn diagram of the number of differentially expressed genes between the two groups. b: Gene ontology analysis of all genes in macrophages cultured on MWCNT vs. nHA scaffolds. BP represents biological process; CC represents cellular component; MF represents molecular function.

10. Cellular uptake of MWCNT by macrophages

To investigate whether MWCNTs could be internalized by cells and whether such uptake was cell-type dependent, RAW264.7 macrophages and rBMSCs were incubated with MWCNT-containing scaffold extracts and examined by transmission electron microscopy (TEM). Electron-dense tubular MWCNT-like structures were readily observed within phagolysosomal vesicles of RAW264.7 cells, whereas no obvious intracellular MWCNT structures were detected in rBMSCs under the same conditions **(Fig. S12)**, suggesting a preferential uptake of MWCNTs by phagocytic macrophages.

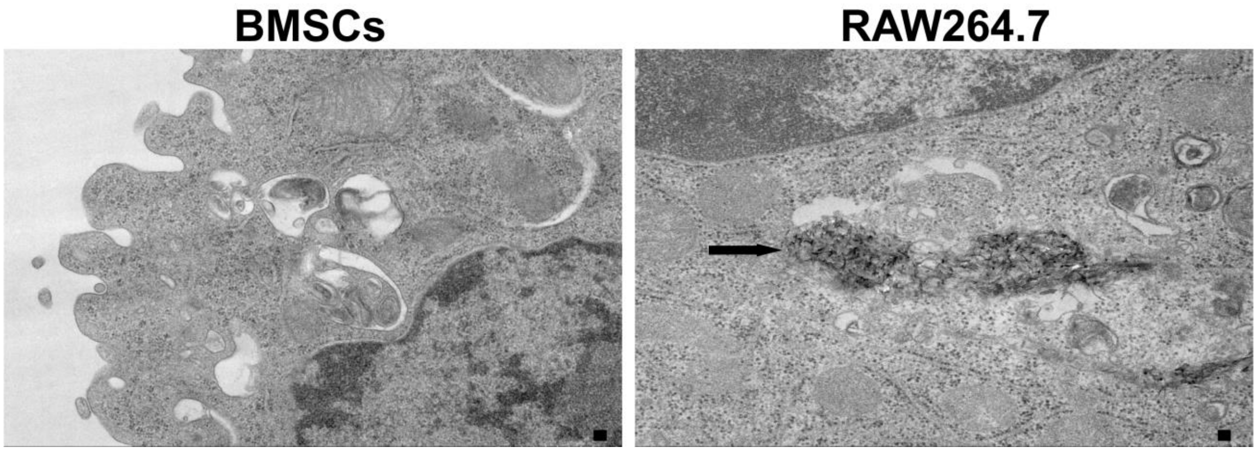

Fig. S12. TEM images showing macrophage internalization of MWCNTs. RAW264.7 macrophages were incubated with extracts of MWCNT scaffolds (1% MWCNT group) for 24 h and processed for TEM observation. Representative images show electron-dense tubular MWCNT-like structures (arrows) within intracellular phagolysosomal vesicles, indicating macrophage phagocytosis of MWCNTs. Scale bar = 500 nm. No obvious intracellular MWCNT structures were observed in BMSCs treated under the same conditions.

11. Relative elemental content of each group of scaffolds.

Table S1. Relative elemental content

nHA	Wt(%)	0.2%MWCNT	Wt(%)	0.5%MWCNT	Wt(%)	1%MWCNT	Wt(%)
C	35.80	C	36.62	C	40.89	C	43.86
O	41.12	O	41.28	O	37.90	O	34.76
P	7.48	P	6.52	P	6.86	P	7.16
Ca	15.60	Ca	15.59	Ca	14.36	Ca	14.22
Total	100.00	Total	100.00	Total	100.00	Total	100.00

12. Primer sequence informationfor RT-qPCR

Table S2. List of Primer Information

Designation	Primer sequence
Arg-1	F: 5' GTAGACAAGCTGGGGATTGG 3'
	R: 5' TCAAAGCTCAGGTGAATCGG 3'
IL-6	F: 5' CAACGATGATGCACTTGCAGA 3'
	R: 5' GTGACTCCAGCTTATCTCTTGGT 3'
IL-10	F: 5' GCTCCAAGACCAAGGTGTCT 3'
	R: 5' CGGAGAGAGGTACAAACGAGG 3'
TNF-α	F: 5' ACGGCATGGATCTCAAAGACA 3'
	R: 5' GTGAGGAGCACGTAGTCGG 3'
BMP2	F: 5' TTTGGCCTGAAGCAGAGACC 3'
	R: 5' ACGGCTTCTTCGTGATGGAA 3'
VEGF	F: 5' GGAGCTTTCACCGAACTCCA 3'
	R: 5' TCTCAGTCCAGGTGAACCGC 3'
iNOS	F: 5' ACCATGAGGCTGAAATCCCA 3'
	R: 5' TCCACAACTCGCTCCAAGAT 3'
MMP9	F: 5' CGCACGACGTCTTCCAGTA 3'
	R: 5' TGCAGGATGTCATAGGTCACG 3'
MMP2	F: 5' CTCCCTGGCTGTTCTGATCG 3'
	R: 5' TGCCAACCCTGTTCCAAAGT 3'
ALP	F: 5' CCGCAGGATGTGAACTACT 3'
	R: 5' GGTACTGACGGAAGAAGGG 3'
Runx2	F: 5' TCGGAAAGGGACGAGAG 3'
	R: 5' TTCAAACGCATACCTGCAT 3'
Collagen I	F: 5' TGCAAGAACAGCGTAGCC 3'
	R: 5' CAGCCATCCACAAGCGT 3'
OPN	F: 5' GAAGCAGTTTACCAAAGATGCCA 3'
	R: 5' AGGGATTTGTTCAGACCTCTCT 3'
GAPDH	F: 5' CAGTGGCAAAGTGGAGATTGTTG 3'
	R: 5' TCGCTCCTGGAAGATGGTGAT 3'
